# Phylogenetic analysis and temporal diversification of the tribe Alsineae (Caryophyllaceae) with the description of three new genera, *Hesperostellaria*, *Reniostellaria* and *Torreyostellaria*

**DOI:** 10.3389/fpls.2023.1127443

**Published:** 2023-06-21

**Authors:** Bine Xue, Zhuqiu Song, Jie Cai, Zhonghui Ma, Jiuxiang Huang, Yuling Li, Gang Yao

**Affiliations:** ^1^ College of Horticulture and Landscape Architecture, Zhongkai University of Agriculture and Engineering, Guangzhou, Guangdong, China; ^2^ Key Laboratory of Plant Resources Conservation and Sustainable Utilization, South China Botanical Garden, Chinese Academy of Sciences, Guangzhou, China; ^3^ Germplasm Bank of Wild Species, Kunming Institute of Botany, Chinese Academy of Sciences, Kunming, Yunnan, China; ^4^ College of Agriculture, State Key Laboratory for Conservation and Utilization of Subtropical Agro-bioresources, National Demonstration Center for Experimental Plant Science Education, Guangxi University, Nanning, China; ^5^ College of Forestry and Landscape Architecture, South China Agricultural University, Guangzhou, China

**Keywords:** Alsineae, Caryophyllaceae, diversification, new combination, new genus, phylogeny

## Abstract

Alsineae are one of the most taxonomically difficult tribes in Caryophyllaceae and consist of over 500 species distributed in the northern temperate zone. Recent phylogenetic results have improved our understanding on the evolutionary relationships among Alsineae members. Nevertheless, there are still some unresolved taxonomic and phylogenetic problems at the generic level, and the evolutionary history of major clades within the tribe was unexplored to date. In this study, we carried out phylogenetic analyses and divergence time estimation of Alsineae using the nuclear ribosomal internal transcribed spacer (nrITS) and four plastid regions (*matK*, *rbcL*, *rps16*, *trnL-F*). The present analyses yielded a robustly supported phylogenetic hypothesis of the tribe. Our results showed that the monophyletic Alsineae are strongly supported to be the sister of Arenarieae, and the inter-generic relationships within Alsineae were mostly resolved with strong support. Both molecular phylogenetic and morphological evidence supported the Asian species *Stellaria bistylata* and the two North American species *Pseudostellaria jamesiana* and *Stellaria americana* all should be recognized as new monotypic genera respectively, and three new genera *Reniostellaria*, *Torreyostellaria*, and *Hesperostellaria* were thereby proposed here. Additionally, molecular and morphological evidence also supported the proposal of the new combination *Schizotechium delavayi*. Nineteen genera were accepted within Alsineae and a key to these genera was provided. Molecular dating analysis suggested that Alsineae splitted from its sister tribe at ca. 50.2 million-years ago (Ma) during the early Eocene and began to diverge at ca. 37.9 Ma during the late Eocene, and divergent events within Alsineae occurred mainly since the late Oligocene. Results from the present study provide insights into the historical assembly of herbaceous flora in northern temperate regions.

## 1 Introduction

**Figure 1:**
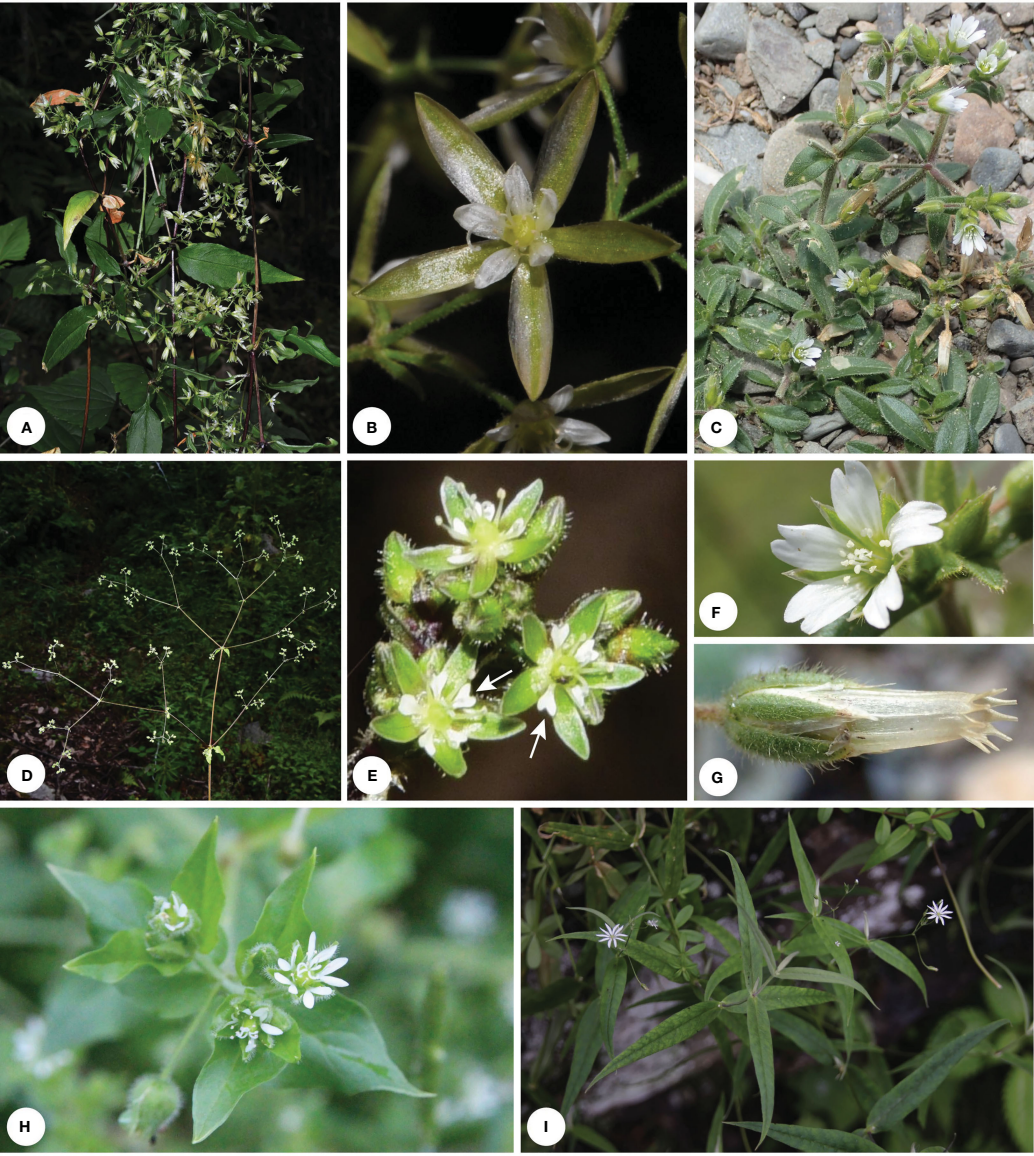
Morphological diversity of some Alsineae species. **(A, B)** *Brachystemma calycinum*. **(C, F, G)** *Cerastium pusillum*. **(D, E)** *Schizotechium paniculatum*; **(H)** *Stellaria aquatica*. **(I)** *Stellaria vestita* var. *amplexicaulis*. Photo by J. Cai **(A, B)**, Z. Ma **(D, E)**, G. Yao **(C, F–I)**.

**Figure 2:**
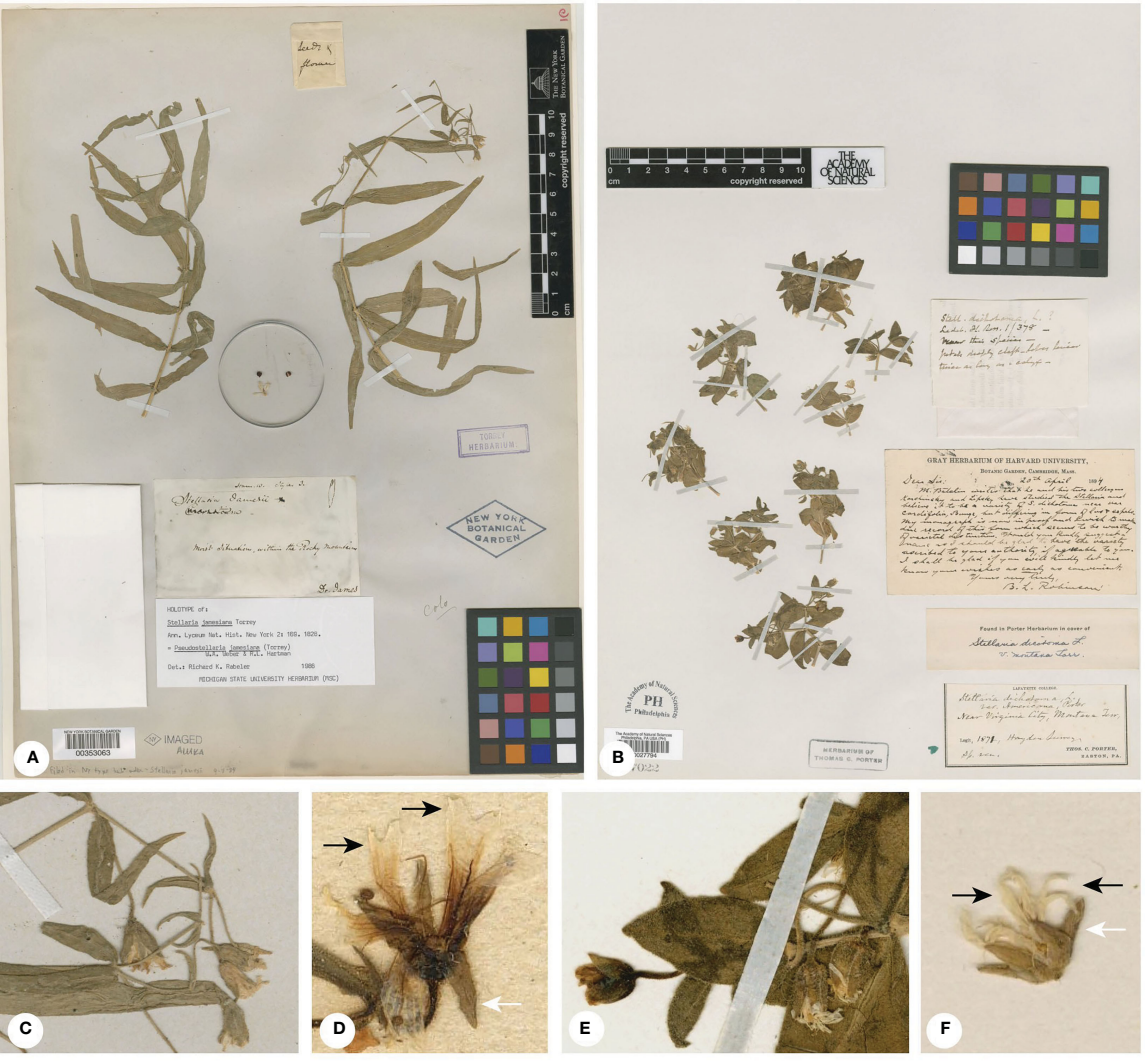
**(A)** Holotype of *Pseudostellaria jamesiana* (=*Torreyostellaria jamesiana*; *E. James s.n.*, NY-00353063); **(B)** Isotype of *Stellaria americana* [=*Hesperostellaria americana*; T. Porter s.n., PH00027794]; **(C)** Inflorescence of *P. jamesiana* from the holotype; **(D)** Flower of *P. jamesiana* (*A.A. Heller 5880*, K000723560); **(E)** Inflorescence of *S. americana* from the isotype; **(F)** Flower of *S. americana* (*T. Porter s.n.*, NY00353059). Black arrowheads indicate petals and white arrowheads indicate sepals.

**Figure 3:**
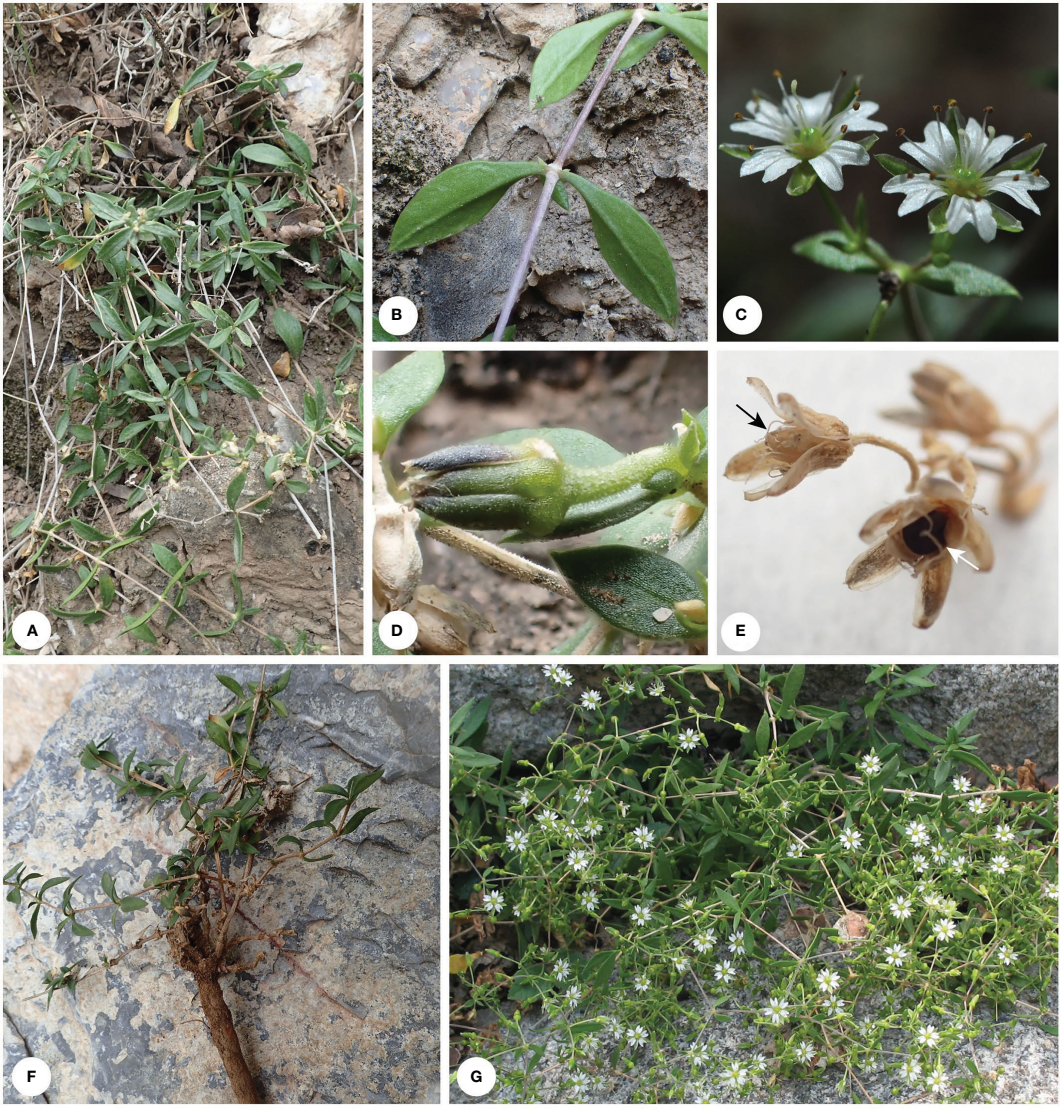
Habit of *Stellaria bistylata*. Photo by G. Yao **(A, B, D–F)** and Q. Zhu **(C, G)**. Black arrowhead indicates capsule and white arrowhead indicates seed in capsule.

Caryophyllaceae are the largest family in the order Caryophyllales, with ca. 100 genera and 3000 species (Hernández-Ledesma et al., 2015). Results of molecular phylogenetic studies have greatly advanced our understanding on the evolutionary relationships among members of the family (Fior et al., 2006; Harbaugh et al., 2010; Greenberg and Donoghue, 2011; Sadeghian et al., 2015). The three traditionally recognized subfamilies within Caryophyllaceae, viz. Alsinoideae, Caryophylloideae and Paronychioideae, were shown to be non-monophyletic, and 12 tribes are now recognized according to recent phylogenetic studies (Harbaugh et al., 2010; Greenberg and Donoghue, 2011; Pusalkar, 2015; Xu et al., 2019). Among them, Alsineae are one of the most taxonomically challenging groups in Caryophyllaceae with over 500 species distributed in the northern temperate zone (Greenberg and Donoghue, 2011; Hernández-Ledesma et al., 2015). The taxonomic history of Alsineae was recently summarized in Arabi et al. (2022), and members of the tribe are usually characterized by their 4–5 free sepals, antesepalous stamens usually with nectary glands at the abaxial base of the filaments, and their distinct styles (**Figures 1**–**3**; Arabi et al., 2022).

In the past two decades, molecular phylogenetic studies have changed our understanding on the circumscription of the tribe Alsineae as well as generic delimitation within it (Fior et al., 2006; Harbaugh et al., 2010; Greenberg and Donoghue, 2011; Dillenberger and Kadereit, 2014; Pusalkar and Singh, 2015; Sadeghian et al., 2015; Zhang et al., 2017; Sharples and Tripp, 2019; Yao et al., 2021; Arabi et al., 2022; Wang et al., 2023). Bittrich (1993) provided a comprehensive taxonomic treatment of Alsineae and accepted 23 genera in the tribe, viz. *Alsinidendron* H. Mann, *Arenaria* L., *Brachystemma* D. Don, *Bufonia* L., *Cerastium* L., *Colobanthus* Bartling, *Holosteum* L., *Honkenya* Ehrh., *Lepyrodiclis* Fenzl, *Minuartia* L., *Moehringia* L., *Moenchia* Ehrh., *Myosoton* Moench, *Plettkea* Mattf., *Pseudostellaria* Pax, *Pycnophyllopsis* Skottsb., *Reicheëlla* Pax, *Sagina* L., *Schiedea* Cham. & Schlchtd., *Stellaria* L., *Thylacospermum* Fenzl, *Thyrya* Boiss. & Bal. and *Wilhelmsia* Reichb. Based on molecular phylogenetic results, however, several genera were removed from Alsineae and placed into other tribes. For instance, *Honkenya*, *Schiedea* (including *Alsinidendron*; Weller et al., 1995; Hernández-Ledesma et al., 2015) and *Wilhelmsia* were transferred to the tribe Sclerantheae (Harbaugh et al., 2010; Greenberg and Donoghue, 2011); *Bufonia*, *Colobanthus* and *Sagina* were moved to Sagineae (Harbaugh et al., 2010; Greenberg and Donoghue, 2011); the genus *Moehringia* and several subgenera of *Arenaria* sensu lato (s.l.) were placed in Arenarieae (Harbaugh et al., 2010; Greenberg and Donoghue, 2011); and the genus *Thylacospermum* was placed in Thylacospermeae (Pusalkar, 2015; Xu et al., 2019). In addition, species of *Minuartia* have been segregated into 11 genera, all of which have been removed from Alsineae (Dillenberger and Kadereit, 2014).

Currently, only two of the ten subgenera of *Arenaria* s.l. remain in the tribe Alsineae and have been elevated to generic level, viz. *Odontostemma* Benth. ex G. Don and *Shivparvatia* Pusalkar & D.K. Singh (Pusalkar and Singh, 2015; Sadeghian et al., 2015). Although the genera *Cerastium*, *Pseudostellaria* and *Stellaria* remain in the newly circumscribed tribe Alsineae, their generic circumscription has been changed. For example, *Cerastium* subg. *Dichodon* (Bartl. ex Rchb.) Boiss. have been resurrected at genus level (Hernández-Ledesma et al., 2015; Yao, 2016), while the Chinese monotypic genus *Pseudocerastium* C.Y. Wu, X.H. Guo & X.P. Zhang was recently revealed as a member of *Cerastium* based on evidence from morphological and molecular data (Yao et al., 2021). Additionally, two North American species of *Pseudostellaria*, i.e. *P. sierrae* Rabeler & R.L. Hartm. and *P. oxyphylla* (B.L. Rob.) R.L. Hartm. & Rabeler, have been segregated as a new genus *Hartmaniella* M.L. Zhang & Rabeler (Zhang et al., 2017). Furthermore, multiple genera, such as *Adenonema* Bunge, *Mesostemma* Vved., *Nubelaria* M.T. Sharples & E. Tripp and *Rabelera* (L.) M.T. Sharples & E. Tripp, have been separated from *Stellaria* s.l. (Sharples and Tripp, 2019), whereas the genera *Plettkea* (= *Pycnophyllopsis*; Timaná, 2017) and *Myosoton* were shown to be nested deeply within the core *Stellaria*, which was circumscribed currently as *Stellaria* sensu stricto (s.s.) (Greenberg and Donoghue, 2011; Yao et al., 2021).

Although great progress has been made in the phylogenetic studies of Alsineae, the generic status of some taxonomic groups within the tribe is still controversial. For example, a well-supported clade consisting of two North American species *Pseudostellaria jamesiana* (Torr.) W.A. Weber & R.L. Hartm. (**Figures 2A, C, D**) and *Stellaria americana* (Porter ex B.L. Rob.) Standl. (**Figures 2B, E, F**) was shown to be distantly related to the core *Pseudostellaria*, core *Stellaria* or *Schizotechium* Rchb. in most previous studies (Greenberg and Donoghue, 2011; Zhang et al., 2017; Sharples and Tripp, 2019; Yao et al., 2021). But Arabi et al. (2022) transferred both species to *Schizotechium* based on the results using two DNA markers (nrITS and *rps16*), which was discordant with the results of previous studies. Although the species in *Stellaria* s.l. have been grouped to multiple genera, some Chinese species (e.g. *S. bistylata* W.Zh. Di & Y. Ren (**Figure 3**) and *S. delavayi* Franch.) that differ from *Stellaria* s.s. in morphology have not been sampled for phylogenetic analyses and their generic affiliation needs to be clarified. In addition, the low support value for some inter-generic relationships is also a common problem in recent phylogenetic studies of Alsineae (Greenberg and Donoghue, 2011; Zhang et al., 2017; Yao et al., 2021). Therefore, increasing taxon sampling in phylogenetic analyses for the tribe Alsineae is essential to clarify the generic affiliation of relevant taxonomic groups. The results would provide important evidences for the taxonomic treatment of the species and genera in Alsineae.

It is hypothesized that the global climate change occurred in different geologic eras usually had a great impact on the evolutionary history of plant groups, such as the climate optimum in the early Eocene may have promoted the diversification of major lineages of Asteraceae (Huang et al., 2016) and Cucurbitaceae (Guo et al., 2020); the warming and aridity climatic condition occurred during the Late Oligocene may have accelerated the rapid diversification of the phaseoloid legumes (Li et al., 2013); and the climate transition from warm and wet condition to cold and dry condition occurred since the mid-Miocene may have triggered the ecological expansion of C_4_ herbs (Sage et al., 2012), and also the development of the global dryland floras (Wu et al., 2019). Caryophyllaceae are a large herbaceous family with most of the species distributed in northern temperate regions, and the tribe Alsineae also represents a large herbaceous lineage mainly distributed in the same regions. In previous dating analyses, however, taxon sampling of Caryophyllaceae mostly focused on several tribes (Gizaw et al., 2016; Jia et al., 2016; Xu et al., 2019) or genera (Frajman et al., 2009; Petri et al., 2013; Mahmoudi-Shamsabad et al., 2021), and the temporal evolutionary history of Caryophyllaceae with a comprehensive tribal level sampling or Alsineae with a comprehensive generic sampling has not been investigated. Exploring the temporal origination and diversification of major clades within these two groups may provide a new case study in understanding the relationship between global climate change and biological evolution, and also would be important to better understand the evolutionary history of herbaceous flora.

In order to better understand the phylogenetic relationships among Alsineae members and explore the temporal evolutionary history of Caryophyllaceae and Alsineae, we newly sequenced some key species of Alsineae in this study, and carried out phylogenetic analyses and divergence time estimation of Caryophyllaceae with the focus on Alsineae. The aim of this study was to (1) improve the resolution of the phylogenetic backbone of Alsineae; (2) clarify the phylogenetic positions and taxonomic status of *Pseudostellaria jamesiana* and *Stellaria americana*, as well as several other Chinese *Stellaria* s.l. species that were not included in previous phylogenetic studies; (3) explore the origination and diversification of major clades within Alsineae and Caryophyllaceae.

## 2 Material and methods

### 2.1 Taxon sampling

Five species [viz. *Adenonema cherleriae* (Fisch. ex Ser.) M.T. Sharples & E.A. Tripp, *Brachystemma calycinum* D. Don, *Schizotechium paniculatum* (Edgew.) Pusalker & S.K. Srivast, *Stellaria bistylata* and *S. delavayi*] belong to four genera of Alsineae were newly sequenced in the present study. We further added sequences of 84 species representing 14 genera of Alsineae from NCBI (https://www.ncbi.nlm.nih.gov/). Thus the tribe Alsineae is extensively sampled according to the previous phylogenetic framework (Zhang et al., 2017; Sharples and Tripp, 2019; Yao et al., 2021), and all the 16 currently accepted genera within Alsineae were included. For the two large genera that both have more than 100 species, viz. *Cerastium* and *Stellaria* s.s., representative species from multiple of their major clades were included based on previous phylogenetic results on the basis of extensive species sampling (Scheen et al., 2004; Sharples and Tripp, 2019; Arabi et al., 2022). In order to use more calibration points within and outside Caryophyllaceae in molecular dating analysis, representatives of all the other 11 tribes currently circumscribed in the family were sampled based on the phylogenetic framework provided previously in Greenberg and Donoghue (2011) and Xu et al. (2019), and representatives of Amaranthaceae, Achatocarpaceae and Macarthuriaceae were selected as outgroups according to the phylogenetic framework of Caryophyllales provided in Yao et al. (2019).

In previous phylogenetic studies, a strategy in order to minimize the proportion of missing data was adopted for some monophyletic genera, with combing sequences from more than one species as the representative molecular data of the genus and then treating these terminal taxa at the generic level in analysis (Baker et al., 2009; Magallón et al., 2015). The same strategy is adopted here for several genera outside Alsineae, in which the congeneric species involved belong to a monophyletic genus or a monophyletic infra-generic clade nested with the genus as referred mainly from the phylogenetic results of Greenberg and Donoghue (2011). Thus, the genera involved in this sampling strategy listed in **Supplementary Material-Table S1** only presented with the generic names.

### 2.2 DNA extraction and sequencing

Total DNA of the silica-gel dried or herbarium material of the five species newly sequenced in the present study were extracted using a modified CTAB method (Doyle and Doyle, 1987), and then sequenced through the genome skimming technique following Zhang et al. (2021). Four plastid regions (*matK*, *rbcL*, *rps16* intron and *trnL-F*) and nrITS were sampled for phylogenetic analyses following the study of Yao et al. (2021). The nuclear ribosomal internal transcribed spacer (nrITS) and plastid sequence reads were thus assembled using the software GetOrganelle (Jin et al., 2020), referenced to the nrITS sequence of *Stellaria media* (L.) Vill. (accession number: MK044722) and plastid genome of *Pseudostellaria heterophylla* (Miq.) Pax (NC_044183), respectively. All of the genes in the plastid genome were annotated using the software PGA (Qu et al., 2019), and the four plastid regions of the currently sequenced species were directly extracted from their plastid genomes assembled here. Sequences newly obtained in the present study have been submitted to the GenBank database, and sequences of other species included in this study were downloaded from the website of NCBI. A few gene sequences of some accessions sampled here were absent and thus coded as missing data. Detailed information about the samples and GenBank accession numbers of all taxon included are listed in **Supplementary Material-Table S1**.

### 2.3 Matrices construction and phylogenetic analyses

Sequences for each DNA region were aligned independently using MAFFT v7.450 (Katoh and Standley, 2013) with the LINSI algorithm in Geneious v.11.0.4 (Kearse et al., 2012). The removal of poorly aligned sites within the *rps16* intron and *trnL-F* intergenic region was conducted in Gblocks 0.91b (Castresana, 2000), with the criterion under ‘Allowed Gap Positions’ set as ‘All’. Then three Caryophyllaceae-wide datasets were constructed: (1) the nrITS dataset, (2) the cpDNA dataset including *matK*, *rbcL*, *rps1*6 and *trnL-F*, (3) and the combined cpDNA-nrITS dataset including all the five DNA regions. Additionally, the *rps16* intron and *trnL-F* intergenic region were much better aligned among Alsineae members compared with those of the above mentioned Caryophyllaceae-wide sampling, thus we also compiled a fourth dataset, i.e. the Alsineae-wide dataset, in which all the Alsineae members were included as ingroups and two species of Arenarieae as outgroups. The Alsineae-wide dataset has the full aligned length of the five DNA regions, because it was not treated with Gblocks but was manually checked, therefore, it would provide more informative DNA sites compared with the results of the Caryophyllaceae-wide analyses. Moreover, considering that all the three individuals of *Mesostemma* only have two DNA markers (nrITS and *rps16*) and thus the genus has a large proportion of missing data in the Alsineae-wide dataset, we excluded this genus from the Alsineae-wide dataset and constructed a fifth matrix, viz. the reduced Alsineae-wide dataset.

Phylogenetic analyses based on the three Caryophyllaceae-wide datasets, the Alsineae-wide dataset and the reduced Alsineae-wide dataset were conducted using Bayesian inference (BI) in MrBayes v.3.2.7 (Ronquist and Huelsenbeck, 2003) and Maximum likelihood (ML) in RAxML-HPC2 v. 8.1.2 (Stamatakis, 2006). Before the conduction of BI analysis, the model of nucleotide substitution for each of the five DNA regions were selected independently under the corrected Akaike Information Criterion (AICc) using jModelTest v.3.7 (Posada, 2008): GTR+I+G for nrITS, GTR+G for *matK*, TIM1+I+G for *rbcL*, GTR+I+G for *rps16* and TPM1uf+G for *trnL-F* were selected for the Caryophyllaceae-wide dataset, while SYM+I+G for nrITS, TPM1uf+G for *matK*, TPM1uf+I+G for *rbcL*, TPM1uf+G for *rps16* and TVM+G for *trnL-F* were selected for both the Alsineae-wide and reduced Alsineae-wide datasets. In BI analysis of the three Caryophyllaceae-wide matrices, the Markov Chain Monte Carlo (MCMC) analysis was run for 20,000,000 generations and sampled every 500 generations. The temperature parameter was set to 0.05 for the combined Caryophyllaceae-wide cpDNA-nrITS dataset, and default for the other two datasets. The parameters used in BI analyses of both the Alsineae-wide and reduced Alsineae-wide matrices followed those used in the BI analysis of the Caryophllaceae-wide cpDNA matrix, except that the MCMC analyses were run for 10,000,000. Number of generations for these datasets were all sufficient, because the effective sample size (ESS) of all parameters were over 200 as evaluated in Tracer v. 1.6 (Rambaut et al., 2014), and the average standard deviations (SD) of split frequencies for each of the datasets were below 0.01. The first 25% of trees obtained in BI analyses were discarded as burn-in and then posterior probabilities (PP) were determined from the posterior distribution. The ML analyses of all the datasets were run on the CIPPRES cluster (Miller et al., 2010) under the GTR+G model, with remaining parameters left at default values, following Yao et al. (2021). A rapid bootstrap (BS) analysis using the same model with 1000 replicates was conducted to obtain the support value for each phylogenetic node. In this study, we defined the high or strong support value as BS ≥ 80% or PP ≥ 0.95, moderate support value as 70% ≤ BS < 80% or 0.90 ≤ PP < 0.95, and low or weak support value as BS < 70% or PP < 0.90, following the definition suggested by Zhao et al. (2021).

### 2.4 Divergence time estimation

Due to the unavailability of fossil record in the tribe Alsineae, the divergence time estimation was conducted under the Caryophyllaceae-wide taxon sampling. Phylogenetic relationships of Caryophyllaceae derived from the combined cpDNA-nrITS dataset were much better resolved than those derived from either the cpDNA or nrITS datasets (referred to the present phylogenetic analyses), thus divergence time estimation is carried out based on the combined Caryophyllaceae-wide cpDNA-nrITS dataset. Two fossil constraints with lognormal distribution prior were used in the present dating analysis: (1) the pollen fossil of *Polyporina cribraria* Srivastava discovered from the stratum of late Cretaceous may represent the earliest known fossil of Amaranthaceae s.l. (including Chenopodiaceae) (Srivastava, 1969; Muller, 1981), thus it is used here to calibrate the crown node of the family as referred from previous studies (Kadereit and Freitag, 2011; Li et al., 2019; Yao et al., 2019), with offset set to 66.0 million-years ago (Ma), a mean of 2.0 and a SD of 1.0; and (2) the inflorescence fossil of *Caryophylloflora paleogenica* G. J. Jord. & Macphail discovered from the stratum of the middle-late Eocene (Jordan and Macphail, 2003) was used widely in previous dating analysis involving Caryophyllaceae members (Frajman et al., 2009; Valente et al., 2010; Smith et al., 2018; Xu et al., 2019; Ding et al., 2020), thus it is used here to calibrate the crown of the “higher” Caryophyllaceae (including the traditionally circumscribed subfamilies Alsinoideae and Caryophylloideae, as defined by Jordan and Macphail, 2003), with offset set to 33.9 Ma, a mean of 2.0 and a SD of 1.0. The option of “*Mean in Real Space*” was checked when setting the ages of the two fossil calibrations. In addition, Yao et al. (2019) carried out divergence time estimation for Caryophyllales based on the well-resolved phylogenetic relationship and multiple fossil calibrations, and the divergence times among Caryophyllaceae and its close relatives were provided in their study. Thus we employed secondary calibration method to constraint another two key nodes according to the dating results of Yao et al. (2019): (1) the stem age of Macarthuriaceae was set as offset = 93.4 Ma, with sigma set to 1.5; and (2) the stem age of Caryophyllaceae was set as offset = 89.0 Ma, with sigma set to 1.5. A normal distribution prior for the age of the two secondary constraints was adopted, as referred from the manual of BEAST software (Bouckaert et al., 2014).

Divergence time estimation was performed using BEAST v. 2.6.6 (Bouckaert et al., 2014) under a lognormal relaxed molecular clock, with Birth-Death speciation process selected as tree prior. The best substitution model was used here for each DNA region based on the above-mentioned result derived from jModelTest v.3.7. MCMC analyses were run 200 million generations, sampled every 2000 generations. Tracer v.1.6 was used to check parameter convergence and adequate effective sample sizes (ESS > 200). TreeAnnotator v.2.6.6 in the BEAST package was used to summarize all the trees based on a target tree derived from ML analysis of the Caryophyllaceae-wide cpDNA-nrITS dataset, with the first 50% of trees discarded as burn-in. The summarized result was visualized in Figtree v.1.4.4 (Rambaut, 2012).

## 3 Results

### 3.1 Phylogenetic analyses

**Figure 4:**
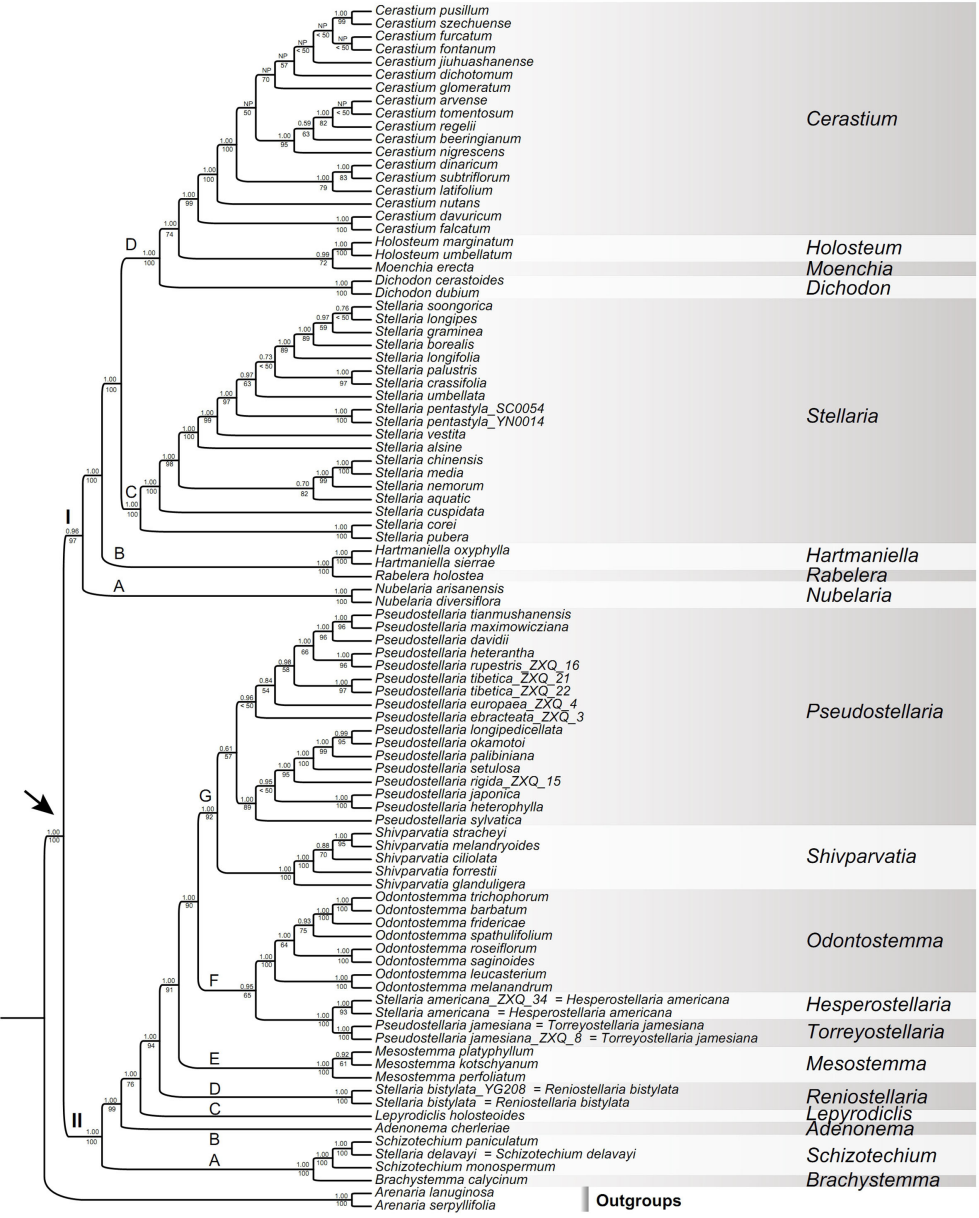
Maximum likelihood (ML) tree of Alsineae inferred from the Alsineae-wide dataset (including nrITS, *matK*, *rbcL*, *rps16* intron and *trnL-F* intergenic region). Posterior probability (PP) in Bayesian inference (BI) and bootstrap (BS) value in ML analysis are indicated above and below the stem branch of each phylogenetic node, respectively. NP indicates the topology was not present in BI analysis. The crown node of Alsineae is shown by the arrowhead.

The cpDNA, nrITS and combined cpDNA-nrITS datasets in Caryophyllaceae-wide taxon sampling contained 3,578 bp, 705 bp and 4,283 bp, respectively. The Alsineae-wide and reduced Alsineae-wide datasets that including all the five DNA regions both contained 4,926 bp. BI and ML analyses of the three Caryophyllaceae-wide datasets yielded largely consistent topologies, except for several nodes conflicted but with low support values (**Figures S1**–**S3**). Such as the tribe Corrigioleae was placed as sister to all the other members of Caryophyllaceae with high supports in analyses of both the cpDNA and combined cpDNA-nrITS datasets (**Figures S2**, **S3**), while the positions of the two tribes Corrigioleae and Paronychieae was interchanged in analysis of the nrITS dataset (**Figure S1**) when compared with those recovered from analyses of the former two datasets, but with low support (**Figure S1**). In Alsineae, phylogenetic positions of several genera (such as *Holosteum*, *Lepyrodiclis*, *Moenchia*, *Nubelaria* and *Shivparvatia*) were conflicted between the phylogenetic results derived from analyses of the nrITS and cpDNA datasets, but all with weak supports (**Figures S1**, **S2**) except the position of *Nuberlaria* was highly supported in analysis of the cpDNA dataset (**Figure S2**). Phylogenetic relationships obtained from analysis of the combined cpDNA-nrITS dataset (**Figure S3**) were resolved with higher supports in most nodes compared with those derived from either the cpDNA or nrITS datasets (**Figures S1**, **S2**). In BI and ML analyses of the Alsineae-wide dataset, we observed the same topology among genera of Alsineae (**Figure 4**) compared with that derived from the combined Caryophyllaceae-wide cpDNA-nrITS dataset (**Figure S3**), but with much higher support values in most nodes. For example, the Alsineae-wide analysis provided stronger support for the sister relationships between *Cerastium* and *Holosteum*-*Moenchia* (BS = 74% and PP = 1.00 vs. BS < 50% and PP = 0.62), between *Holosteum* and *Moenchia* (BS = 72% and PP = 0.99 vs. BS <50% and PP = 0.73), between the genus *Adenonema* and its sister (BS = 99% and PP = 1.00 vs. BS = 59% and PP= 1.00), between *Lepyrodiclis* and its sister (BS = 76% and PP = 1.00 vs. BS < 50% and PP= 0.80), between *Stellaria americana*-*Pseudostellaria jamesiana* and *Odontostemma* (BS = 65% and PP = 0.95 vs. BS < 50 and PP= 0.70), between (*Stellaria americana*-*Pseudostellaria jamesiana*)-*Odontostemma* and the core *Pseudostellaria*-*Shivparvatia* (BS = 90% and PP = 1.00 vs. BS = 74% and PP = 1.00), and between the core *Pseudostellaria* and *Shivparvatia* (BS = 92% and PP = 1.00 vs. BS = 75% and PP= 0.97). Additionally, compared with the phylogenetic results derived from the Alsineae-wide dataset (**Figure 4**), analyses of the reduced Alsineae-wide dataset yielded a consistent topology with several nodes better supported (**Figure S4**), such as the stem nodes of *Lepyrodiclis* (BS = 86% and PP = 1.00 vs. BS = 76% and PP= 1.00), *Odontostemma* (BS = 83% and PP = 0.99 vs. BS = 65% and PP = 0.95), and (*Stellaria americana*-*Pseudostellaria jamesiana*)-*Odontostemma* (BS = 97% and PP = 1.00 vs. BS = 90% and PP = 1.00). Here, we focus on describing the phylogenetic relationships among Alsineae members based on the phylogenetic results derived from the Alsineae-wide dataset (**Figure 4**), because most nodes in phylogenetic trees obtained from this dataset were resolved with high supports and this dataset also includes more generic sampling compared with that of the reduced Alsineae-wide dataset.

The monophyly of Alsineae was highly supported (BS = 100%, PP = 1.00; **Figures 4**; **S3**, **S4**). Two major clades were recovered within Alsineae (**Figure 4**): clade I containing eight genera [viz., *Cerastium*, *Dichodon* (Bartl. ex Rchb.) Rchb., *Hartmaniella*, *Holosteum*, *Moenchia*, *Nubelaria*, *Rabelera* and *Stellaria* s.s.], and clade II containing eight genera [viz., *Adenonema*, *Brachystemma*, *Lepyrodiclis*, *Mesostemma*, *Odontostemma*, *Pseudostellaria*, *Schizotechium* and *Shivparvatia*] and three species of *Stellaria* s.l. (viz. *S. americana*, *S. bistylata* and *S. delavayi*). Within clade I, the genus *Nubelaria* (subclade I-A) was sister to the remaining members of this clade (BS = 97%, PP = 0.96), and then followed by the well supported *Hartmaniella*-*Rabelera* subclade (subclade I-B; BS = 100%, PP = 1.00). *Stellaria* s.s. (subclade I-C; BS = 100%, PP = 1.00) was sister to the subclade I-D (BS = 100%, PP = 1.00), the latter of which includes the other four genera (viz., *Cerastium*, *Dichodon*, *Holosteum* and *Moenchia*). Relationships among these four genera were moderately supported in ML analysis but highly supported in BI analysis, such as the sister relationship between *Holosteum* and *Moenchia* (BS = 72%, PP = 0.99), and between *Cerastium* and the *Holosteum*-*Moenchia* group (BS = 74%, PP = 1.00).

Within clade II, a highly supported subclade including *Brachystemma*, *Schizotechium* and *Stellaria delavayi* (subclade II-A; BS = 100%, PP = 1.00) was sister to the rest of the clade, and then followed successively by *Adenonema* (subclade II-B), *Lepyrodiclis* (subclade II-C), *Stellaria bistylata* (subclade II-D), and *Mesostemma* (subclade II-E). The relationships among these subclades were strongly supported (BSs > 90%, PPs = 1.00) except the stem node of *Lepyrodiclis* moderately supported in ML analysis (BS = 76%), but this node was strongly supported in analysis of the reduced Alsineae-wide dataset (BS = 86%, PP = 1.00; **Figure S4**). The subclade II-F comprising the genus *Odontostemma* and *Stellaria americana*-*Pseudostellaria jamesiana* was lowly supported in ML analysis (BS = 65%) but highly supported in BI analysis (PP = 0.95), and also highly supported in analysis of the reduced Alsineae-wide dataset (BS = 83%, PP = 0.99; **Figure S4**). Additionally, within clade II, the seventh subclade (II-G) comprising *Pseudostellaria* (except *P. jamesiana*) and *Shivparvatia* were also strongly supported in both ML and BI analyses (BS = 92%, PP = 1.00).

### 3.2 Divergence time estimation

**Figure 5:**
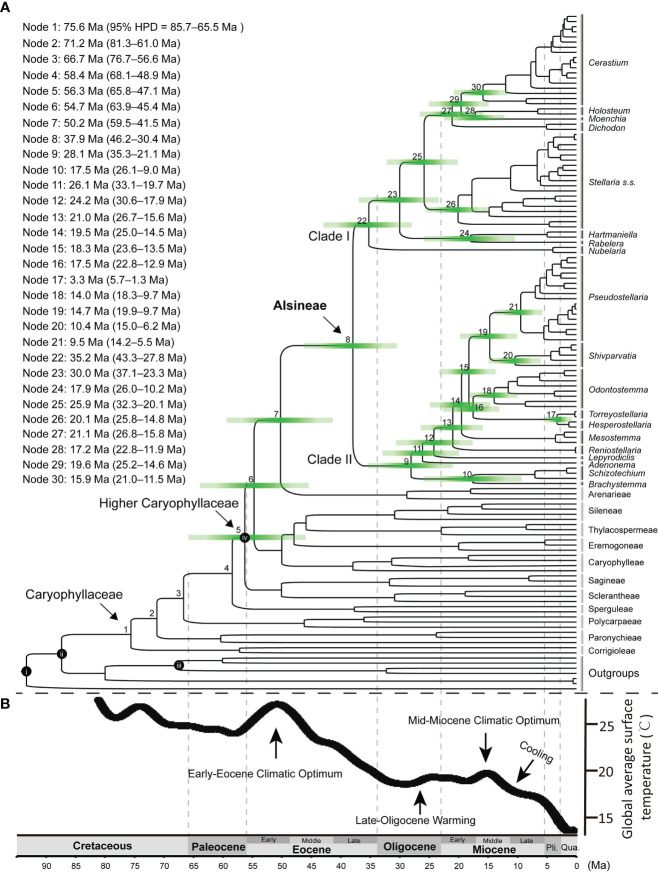
**(A)** Chronogram using the Caryophyllaceae-wide cpDNA-nrITS dataset (including nrITS, *matK*, *rbcL*, *rps16* intron and *trnL-F* intergenic region) and four calibration points (i–iv), i: stem node of Macarthuriaceae, ii: stem node of Caryophyllaceae, iii: crown node of Amaranthaceae s.l., iv: crown node of the higher Caryophyllaceae; Green bars represent 95% credibility interval for phylogenetic node, time scale is shown at the bottom of the figure; Divergence ages for each node marked on the tree are provided at the upper left of the figure. **(B)** The global climate curve since the late Cretaceous, modified from Tierney et al. (2020). Major climate events were indicated following Zhou et al. (2022). Pli., Pliocene; Qua., Quaternary; Ma, Million-years ago.

The result revealed that the family Caryophyllaceae began to diversify at ca. 75.6 Ma [node 1 in **Figure 5**; 95% highest posterior density (HPD) = 85.7–65.5 Ma] in the late Cretaceous, and most tribes in the family split from their sister clade during the early to middle Eocene (**Figure 5**). Rapid radiations near or within the early Eocene (from 56.3 Ma to 54.7 Ma in nodes 5 and 6) may have occurred in the early evolution of “higher Caryophyllaceae”, which includes the tribes except for the four early diverged ones (viz., Corrigioleae, Paronychieae, Polycarpaeae and Sperguleae). The split between Alsineae and its sister Arenarieae occurred at ca. 50.2 Ma (59.5–41.5 Ma) in the early Eocene (node 7), and Alsineae started to diversify at ca. 37.9 Ma (46.2–30.4 Ma) in the late Eocene (node 8). For the two major clades in Alsineae, clade I started to diversify at ca. 35.2 Ma (43.3–27.8 Ma) near the Eocene-Oligocene boundary (node 22), and clade II started to diversify at ca. 28.1 Ma (35.3–21.1 Ma) in the Late Oligocene (node 9). Diversification events for most genera of Alsineae and major lineages of some large genera in the tribe mostly occurred since or near the middle Miocene (**Figure 5**), such as the splits between *Holosteum* and *Moenchia* [ca. 17.2 Ma (22.8–11.9 Ma); node 28], between *Hartmaniella* and *Rabelera* [ca. 17.9 Ma (26.0–10.2 Ma); node 24], between *Pseudostellaria* and *Shivparvatia* [ca. 14.7 Ma (19.9–9.7 Ma); node 19], between *Brachystemma* and *Schizotechium* [ca. 17.5 Ma (26.1–9.0) Ma; node 10], the crown of *Cerastium* [ca. 15.9 Ma (21.0–11.5 Ma); node 30], and the crown of *Odontostemma* [ca. 14.0 Ma (18.3–9.7 Ma); node 18], as well as major lineages of the core *Stellaria* (**Figure 5**).

## 4 Discussion

### 4.1 Improved phylogenetic resolution of the tribe Alsineae

Alsineae are a large and taxonomically challenging tribe in Caryophyllaceae, both in traditional taxonomy period and under the molecular phylogenetic background. Greenberg and Donoghue (2011) provided an important phylogenetic framework for the tribe. Since then, more and more phylogenetic research has been conducted on Alsineae, and the phylogenetic positions and relationships of more and more Alsineae members have been clarified (See ‘Introduction’). Compared with the results of previous studies, our analyses resulted in similar phylogenetic relationships among the Alsineae members, but with higher support values at most nodes.

In the present study, a robust phylogeny of Alsineae with the complete sampling of all currently accepted genera within the tribe was provided, with relationships among these genera highly supported in both BI and ML analyses (**Figures 4**, **S4**), except that the sister relationships between *Holosteum* and *Moenchia*, and between *Cerastium* and *Holosteum*-*Moenchia* obtained moderate supports in ML analysis (BS = 72% and BS = 74% respectively, in **Figure 4**; BSs = 77% in **Figure S4**). Compared with the ML result of Greenberg and Donoghue (2011), the support values at some key nodes within Alsineae were resolved with higher support in our study. For example, the support value for the crown node of clade I increased from BS = 66% in Greenberg and Donoghue (2011) to BS = 97% (**Figure 4**), and the support value for the subclade I-D (*Cerastium*-*Holosteum*-*Dichodon*-*Moenchiac* group) also increased from BS = 87% in Greenberg and Donoghue (2011) to BS = 100% (**Figure 4**). Within clade II, phylogenetic positions of the two genera *Lepyrodiclis* and *Schizotechium* were not resolved or weakly supported in previous studies (Greenberg and Donoghue, 2011; Sadeghian et al., 2015; Zhang et al., 2017; Yao et al., 2021; Arabi et al., 2022), while in our results, phylogenetic positions of the two genera were both well resolved with high supports, especially in analysis of the reduced Alsineae-wide dataset (BSs ≥ 86%, PPs = 1.00; **Figure S4**). Additionally, the sister relationships between *Odontostemma* and the *Pseudostellaria jamesiana*-*Stellaria americana* lineage, between *Pseudostellaria* (except *P. jamesiana*) and *Shivparvatia*, as well as the sister relationship between the two subclades II-F and II-G, were not resolved or lowly supported in previous studies (Greenberg and Donoghue, 2011; Sadeghian et al., 2015; Zhang et al., 2017; Yao et al., 2021; Arabi et al., 2022; Wang et al., 2023), but were well resolved with high supports in our analyses (**Figures 4**, **S4**). On the other hand, the generic relationships within Alsineae obtained here (**Figure 4**) are highly congruent with those reported in Sharples and Tripp (2019), which was conducted on the basis of reduced representation genomic data but with fewer generic sampling of Alsineae.

Previous studies have highlighted the importance of broad taxon sampling in improving the accuracy of phylogenetic inference, because inclusion of more taxa may be useful in improving the detection of multiple substitutions to some extent (Wiens, 2005; Xi et al., 2012; Yao et al., 2019). This viewpoint was also supported in the present study. The improvements gained here in terms of resolution and support of the phylogeny of Alsineae may be attributed to the more extensive generic sampling compared with previous studies, which were conducted on the basis of the same or similar set of DNA markers compared with the present study but with fewer generic sampling of Alsineae (e.g., Greenberg and Donoghue, 2011; Zhang et al., 2017; Yao et al., 2021; Wang et al., 2023). Such as compared with the results of Yao et al. (2021) and Wang et al. (2023), in which the same set of DNA markers were analyzed but fewer genera of Alsineae were included, phylogenetic positions of multiple genera within the tribe were better resolved and/or obtained higher supports in the present study, including *Holosteum*, *Lepyrodiclis*, *Moenchia* and *Shicparvatia* (**Figures 4**; **S4**). Additionally, including more taxa, even though those with a large proportion of missing data, was also suggested to be important to improve the phylogenetic resolution (Xi et al., 2012; Yao et al., 2019; Yao et al., 2023). In the present study, the phylogenetic positions of representatives of some genera that have large proportion of missing data were well-resolved with high support values, especially for the genus *Mesostemma*. Although only two markers (ITS and *rps16* intron) of *Mesostemma* were available for our analyses, phylogenetic position of this genus was well resolved with high support (**Figure 4**) and also consistent with that derived from analysis based on genomic data (Sharples and Tripp, 2019), indicating that the limited molecular data used here may be enough to accurately place the genus on the phylogenetic tree. However, it noteworthy that, including *Mesostemma* with limited sequences here resulted in the reduction of support for several other nodes near the stem node of *Mesostemma*, especially the placement of the *Pseudostellaria jamesiana*-*Stellaria americana* lineage as compared with the result from analysis of the reduced Alsineae-wide matrix (**Figure S4**). Although a robust phylogenetic framework of the tribe Alsineae was provided in the present study, an increase of species and DNA markers (especially the genomic data) is still necessary to better understand the phylogenetic relationships among the Alsineae members.

### 4.2 Generic placements of *Pseudostellaria jamesiana* and *Stellaria americana*

The generic affiliation of the North American species *Pseudostellaria jamesiana* (**Figures 2A, C, D**) is a controversial issue in taxonomy, which have been previously placed in three different genera, viz. *Arenaria*, *Pseudostellaria* and *Stellaria* (Weber and Hartman, 1979). Morphologically, the species has 3 styles, 10 stamens and 6-valved capsules, which supported its inclusion within *Stellaria* (as *S. jamesiana* Torr.). While Shinners (1962) noticed that the petal of *S. jamesiana* is shallowly lobed with a U-shaped sinus at the apex (**Figure 2D**), which seems to be much different from that of *Stellaria* but similar to some species of *Arenaria*, thus the combination *A. jamesiana* (Torr.) Shinners was proposed. After detailed morphological investigation, Weber and Hartman (1979) suggested that the petal morphology observed in *S. jamesiana* is also possessed by the genus *Pseudostellaria*, and the tuberous root and the possession of many barren flowers of *S. jamesiana* may further indicate its affiliation with *Pseudostellaria*. Thus the species *S. jamesiana* was transferred to *Pseudostellaria* accordingly (Weber and Hartman, 1979). Recently, the phylogenetic analysis by Greenberg and Donoghue (2011) revealed a highly supported sister relationship between *Pseudostellaria jamesiana* and another North American species *Stellaria americana* (**Figures 2B, E, F**), with the collective clade located distinct from either the core *Pseudostellaria* or the core *Stellaria*. The relationship was further supported in subsequent phylogenetic studies (Zhang et al., 2017; Yao et al., 2021), although the position of *Pseudostellaria jamesiana*-*Stellaria americana* was lowly supported. In Sharples and Tripp’s (2019) phylogenetic analysis based on reduced representation genomic data, the *Pseudostellaria jamesiana*-*Stellaria americana* lineage also was highly supported and it was sister to the core *Pseudostellaria* (only one species was sampled) with strong support, however other genera closely related to the core *Pseudostellaria* (viz. *Odontostemma* and *Shivparvatia*) were not sampled in their study. Nevertheless, all the above-mentioned studies didn’t clarify the generic affiliation of *Pseudostellaria jamesiana* and *Stellaria americana*.

In the present study, we used two accessions from GenBank for each of *Pseudostellaria jamesiana* and *Stellaria americana*, and found that the monophyly of both species and their sister relationship are all strongly supported (BSs ≥ 93%, PPs = 1.00; **Figure 4**). Furthermore, the sister relationship between *Odontostemma* and *Pseudostellaria jamesiana*-*Stellaria americana*, which had been lowly supported in Greenberg and Donoghue (2011), was also supported in the present ML and BI analyses (BS = 65%, PP = 0.95; **Figure 4**), and highly supported in analyses of the reduced Alsineae-wide dataset (BS = 83%, PP = 0.99; **Figure S4**). Our results also showed that the *Pseudostellaria jamesiana*-*Stellaria americana* lineage is distantly related to the Asian genus *Schizotechium* (**Figure 4**), which is consistent with phylogenetic results reported previously based on analyses of either multiple DNA fragments (Greenberg and Donoghue, 2011; Zhang et al., 2017; Yao et al., 2021) or reduced representation genomic data (Sharples and Tripp, 2019), but is in disagreement with the most recent report in Arabi et al. (2022). It is noteworthy that, the DNA sequences (nrITS and *rps16* intron) of both *Pseudostellaria jamesiana* and *Stellaria americana* used in Arabi et al. (2022) were sequenced mainly by Greenberg and Donoghue (2011) and Zhang et al. (2017). However, the sister relationship between *Pseudostellaria jamesiana*-*Stellaria americana* and *Schizotechium* revealed in Arabi et al. (2022) is inconsistent with those of the phylogenetic analyses using these sequences in Greenberg and Donoghue (2011), Zhang et al. (2017), Yao et al. (2021) and the present study (**Figures 4**, **S4**), although the cause of the conflict between Arabi et al. (2022) and other above-mentioned studies with respects to the phylogenetic position of *Pseudostellaria jamesiana*-*Stellaria americana* is unclear.

Morphologically, the core *Pseudostellaria* native to Asia is characterized by having both chasmogamous and cleistogamous flowers, 3-valved capsules and small chromosome number (2n = 32) (Choi and Pak, 1999; Lu et al., 2001), while *Pseudostellaria jamesiana* has only chasmogamous flowers, 6-valved capsules and much larger chromosome number (2n = 96) (Rabeler and Hartman, 2005). The core *Stellaria* is characterized by having 10 stamens, 6-valved capsules (except for *S. aquatic* and *S. pentastyla* with 5-valved capsule) and usually numerous seeds per capsule (Lu et al., 2001; Sharples and Tripp, 2019), but *Stellaria americana* is characterized in having 5 stamens, 3-valved capsules and only 3–6 seeds per capsule (Rabeler and Hartman, 2005). Thus, the morphological evidence showed that the two species *Pseudostellaria jamesiana* and *Stellaria americana* can be readily distinguished from the core *Pseudostellaria* and core *Stellaria*, which is consistent with the results from molecular analyses (**Figures 4**; **S4**).

Arabi et al. (2022) suggested that their newly circumscribed genus *Schizotechium* (including *Pseudostellaria jamesiana* and *Stellaria americana*) is characterized by having perennial tuberous or stoloniferous herbs, 4-angled stems, 3 styles, 1–6-seed capsules, seeds are 2.5–3.5 mm in diameter and slightly beaked inwards at the apex. However, their treatment to enlarge the genus *Schizotechium* brought more problems for the taxonomy of Alsineae. Firstly, the above-mentioned characters could not be used to distinguish the newly defined *Schizotechium* easily from other genera of Alsineae, especially from the genus *Brachystemma* that was recovered here to be the sister of *Schizotechium* although which was not sampled in Arabi et al. (2022). Because the above-mentioned characters could be also observed in *Brachystemma* except its 2 styles. But the genus *Schizotechium* defined by Pusalkar and Srivastava (2016) actually has 2–3 styles. Secondly, their newly defined *Schizotechium* represents a genus with high morphological heterogeneities, and its generic morphological description should be revised to a large extent compared with that provided by Pusalkar and Srivastava (2016). As referred from Pusalkar and Srivastava (2016), the traits including the large size of plants (**Figure 1D**), much branched and sub-scandent stems, large panicle formed by many-flowered cymes (**Figure 1D**), petals much shorter than sepals and 2-lobed for more than 1/3 to nearly up to the base (**Figure 1E**) are good diagnostic characters to distinguish *Schizotechium* from all the other genera in Alsineae. However, the two species *Pseudostellaria jamesiana* (**Figures 2A, C, D**) and *Stellaria americana* (**Figures 2B, E, F**) are much different from the genus *Schizotechium* defined by Pusalkar and Srivastava (2016) by the smaller size of plants (**Figures 2A, B**), non-scandent stems (**Figures 2A, B**), few-flowered cymes not forming panicles (**Figures 2C, E**), petals much longer than sepals (**Figures 2D, F**). Thus, we suggest that it is not reasonable to include the two species *Pseudostellaria jamesiana* and *Stellaria americana* within *Schizotechium*.

On the other hand, although the two North American species *Pseudostellaria jamesiana* and *Stellaria americana* have some similarities in morphology, such as 4-ridged stems, inflorescence cymes not forming panicle (**Figures 2C, E**), petals much longer than sepals (**Figures 2D, F**) and 3 styles (Rabeler and Hartman, 2005), there are more differences between them. *Pseudostellaria jamesiana* has tuberous root, erect or ascending stems that are usually 12–45 (–60) cm long (**Figure 2A**), leaf blades that are linear to broadly lanceolate in shape (**Figure 2A**) and usually 2–10 cm long and 0.2–1.5 cm wide in size (**Figure 2A**), open cymes (**Figure 2C**) with flowers often proliferating with age, petals that are shallowly 2-lobed at apex (**Figure 2D**), 10 stamens, 6-valved capsules, 1–3 seeds per capsule, and seeds that are reddish brown and broadly elliptic in shape (Rabeler and Hartman, 2005). In contrast, *Stellaria americana* has rhizomatous rootstocks, spreading stems 10–20 cm long and forming prostrate mats (**Figure 2B**), leaf blades that are ovate to lanceolate-ovate in shape (**Figures 2B, E**), and usually 0.8–3 cm long and 0.2–1.3 cm wide in size, very leafy cymes with 1–5 flowers (**Figure 2B**), petals that are deeply 2-lobed at apex (**Figure 2F**), 5 stamens, 3-valved capsules, 3–6 seeds per capsule, and seeds that are rusty brown and sub-ovate in shape (Rabeler and Hartman, 2005). Furthermore, *Stellaria americana* is distinct from all the other members of Alsineae by its tortuous fruiting pedicels that could push the opening capsule with its seeds into the substrate (Rabeler and Hartman, 2005).

As suggested previously, the presence or absence of tuberous root, the pattern of inflorescence, the number of styles, stamens, valves of capsule and seeds per capsule are important characters for generic delimitation in Alsineae (Lu et al., 2001; Zhang et al., 2017; Yao et al., 2019). The two species *Pseudostellaria jamesiana* and *Stellaria americana* differ significantly from each other and also from either the core *Pseudostellaria* or core *Stellaria* in the above-mentioned characters. More detailed morphological comparison among *Pseudostellaria jamesiana*, *Stellaria americana* and other relevant genera is presented in **Table 1** and **Box 1**. Therefore, we propose that the two North American species *Pseudostellaria jamesiana* and *Stellaria americana* should be treated as two distinct genera based on the morphological and molecular evidence. Until now, no generic names had been used for the two species. Therefore, new generic names for the two species should be proposed accordingly. It worth noted that, the acceptance of *Pseudostellaria jamesiana* and *Stellaria americana* as two independent genera is not conflict with the phylogenetic result of Arabi et al. (2022), in which the clade comprised by the two species was also outside the genus *Schizotechium* that was circumscribed by Pusalkar and Srivastava (2016).

**Table 1:** Morphological comparison among *Hesperostellaria*, *Torreyostellaria* and relevant genera.

Character	*Brachystemma*	*Hesperostellaria* (*Stellaria americana*)	*Pseudostellaria*	*Schizotechium*	*Stellaria*	*Torreyostellaria* (*Pseudostellaria jamesiana*)
**Habit**	Annual	Perennial	Perennial	Perennial	Annual, biennial or perennial	Perennial
**Stems**	Diffuse or climbing among shrubs, up to 6 m long	10–20 cm long; spreading and forming loose, prostrate mats;	Erect or ascending, sometimes repent;	Usually much longer than 1 m; sub-scandent	Erect, ascending or prostrate	12–45(-60) cm long; Erect or ascending;
**Root**	Unknown	Rhizomatous rootstocks	Tuberous	Tuberous or thick, fleshy	Taproots usually slender, perennial taxa often rhizomatous;	Tuberous
**Inflorescence**	Many-flowered thyrsi (cymes forming large panicle)	Very leafy cymes, 1–5-flowered	Cymes not forming panicle, or solitary flower in distal leaf axils	Many-flowered compounds cymes forming terminal, large panicle	Solitary flower, or cymes but not forming panicle	Cymes, with flowers often proliferating with age, but not forming panicle
**Flower**	Chasmogamous; bisexual and functional female (gynodioecious);	Chasmogamous; bisexual	Chasmogamous and cleistogamous; bisexual;	Chasmogamous; bisexual;	Chasmogamous; bisexual	Chasmogamous; bisexual;
**Petal**	Much shorter than sepals; margin entire;	Much longer than sepals; deeply 2-lobed at apex;	Longer than sepals; entire or emarginated;	Shorter than sepals; 2-lobed for more than 1/3 to nearly up to the base;	Usually longer than sepals; deeply lobed at apex;	Much longer than sepals; shallowly 2-lobed at apex;
**Style**	2	3	3 (2)	3 (2)	3 (5)	3
**Stamen**	5; filaments much shorter than sepals and not exserted from flowers;	5; filaments longer than sepals and usually exserted from flower;	10 (8); filaments longer than sepals and usually exserted from flowers;	5 or 10; filaments much shorter or rarely sub-equivalent to sepals in length;	10; filaments usually exserted from flowers;	10; filaments longer than sepals and usually exserted from flowers;
**Capsule**	4-valved	3-valved	3 (rarely 2)-valved	6-valved	6 (5)-valved	6-valved
**Seed**	1 per capsule; reniform or globose; testa tuberculate;	3–6 per capsule; Ovate; testa finely tuberculate;	Less than 10 per capsule; somewhat flattened; testa tuberculate or smooth;	1–2 per capsule; Sub-orbicular to sub-reniform; testa wrinkled, rugose or reticulate;	Numerous per capsule; usually reniform, slightly compressed; testa tuberculate or smooth	1–3 per capsule; broadly elliptic; testa tubercles conic to elongate, rounded; ± plump;

Information about the flower of *Pseudostellaria* is described from its chasmogamous flowers.

Box 1 **Key to the genera of Alsineae (modified from Sharples and Tripp, 2019; Arabi et al., 2022).**

1. Fruits capsules; a few seeds (1–10) per capsule.................................................................................................................................................................................................................2

1. Fruits capsules; many seeds (more than 10) per capsule; if fruit a utricle, then 1-seeded......................................................................................................................................................14

2. Capsule opening with as many teeth as styles.................................................................................................................................................................................*Lepyrodiclis*

2. Capsule opening with twice as many teeth as styles.........................................................................................................................................................................................................3

3. Roots tuberous..................................................................................................................................................................................................................................................................................................................4

3. Taproot, or rhizomatous plants with fibrous roots, never tuberous or woody at the base and underground.......................................................................................................6

4. Cleistogamous flowers present ...................................................................................................................................................................................................................*Pseudostellaria*

4. Cleistogamous flowers absent ..............................................................................................................................................................................................................................................................................................................5

5. Cymes not forming panicle; petals much longer than sepals, 2-lobed for less than 1/3; stamens 10; native to North America..............................................*Torreyostellaria*

5. Cymes forming large panicle; petals shorter than sepals, 2-lobed for more than 1/3 to nearly up to the base; stamens 5 or 10; native to Asia.................................................................................................................................................................................................................................................................................*Schizotechium*

6. Stamens 5; native to North America...................................................................................................................................................................................................................................7

6. Stamens 10; native to Asia.............................................................................................................................................................................................................................................................................................8

7. Leaves ovate to ovate-lanceolate; capsules 3–valved; seeds 3–6......................................................................................................................................................... *Hesperostellaria*

7. Leaves lanceolate or elliptic; capsules 6-valved; seeds 1–2........................................................................................................................................................................*Hartmaniella*

8. Sepals saccate; seeds winged .......................................................................................................................................................................................................................*Odontostemma*

8. Sepals non-saccate; seeds wingless ..................................................................................................................................................................................................................................................................9

9. Petals entire at apex .............................................................................................................................................................................................................................................................10

9. Petals emarginate to lobed to 1/2 or more of petal length or absent ..........................................................................................................................................................................11

10. Perennial, laxly caespitose herbs; dwarf, 2–8 cm high; flowers solitary, rarely in pairs, terminal; petals 1.5 to 2 times as long as sepals; styles 2 or 3 .................................................................................................................................................................................................................................................................................................................*Shivparvatia*

10. Annual, sub-scandent herbs; stems up to 6 m long; inflorescence a thyrsi (paniculate cymes), terminal or axillary; petals sub-equivalent as long as sepals or much shorter than sepals; styles 2........................................................................................................................................................................................................................................*Brachystemma*

11. Stems terete; styles usually 2; capsules usually 4-valved; petals 2-lobed to the middle..........................................................................................................................................12

11. Stems terete or not; styles 3; capsules 6-valved; petals deeply lobed or minute/absent.........................................................................................................................................13

12. Inflorescences dichotomous cymes; flowers 5-merous; capsules obovoid; seeds tuberculate...........................................................................................................*Reniostellria*

12. Inflorescences paniculate-dichasial; flowers 4-merous; capsules ovoid; seeds corrugate....................................................................................................................................................*Mesostemma*

13. Stems 4-angled, slender; rhizomes creeping; leaves 3–11.5 long and 4–12 mm wide; petals much longer than sepals; capsules approximately equaling the sepals*............................................................................................................................................................................................................................................................................Rabelera*

13. Stems ± circular in cross-section; woody at the base and underground; leaves less than 3 cm long and 4 mm wide; petals much shorter than sepals; capsules usually shorter than the sepals............................................................................................................................................................................................................................................……*Adenonema*

14. Capsule globose or conic, or fruit indehiscent (in the former *Plettkea* spp.)...................................................................................................................................................15

14. Capsule cylindrical ............................................................................................................................................................................................................................................................16

15. Petals deeply notched, rarely absent.....................................................................................................................................................................................................................*Stellaria*

15. Petals not deeply notched, 2-lobed near apex.................................................................................................................................................................................................*Nubelaria*

16. Cymes umbellate; stamens 3–5; seeds shield-shaped...................................................................................................................................................................................*Holosteum*

16. Cymes not umbellate or weakly so; stamens 4 or 10; seeds not shield shaped........................................................................................................................................................17

17. Styles 3; capsules 6-valved ..................................................................................................................................................................................................................................*Dichodon*

17. Styles 4 or 5; capsules 8- or 10-valved ............................................................................................................................................................................................................................18

18. Sepals 4; petals 4 or rarely absent; styles 4; stamens 4; capsules 8-valved; annuals; petals entire or scarcely retuse at apex .................................................................................................................................................................................................................................................................................................*Moenchia*

18. Sepals 5; petals 5; styles 5; stamens 10; capsules 10-valved; annuals or perennials; petals emarginate or bifid at apex, rarely absent ......................................*Cerastium*

### 4.3 Taxonomic treatment of Chinese *Stellaria* s.l. species

*Stellaria* s.l. is a large genus in the tribe Alsineae with ca. 150–200 species mainly distributed from Eurasia to North America (Hernández-Ledesma et al., 2015). It is one of the most taxonomically difficult genera in Caryophyllaceae because of its highly morphological heterogeneity. There are more than 60 species of *Stellaria* s.l. recorded in China (Wu and Ke, 1996; Lu et al., 2001). Wu and Ke (1996) divided Chinese *Stellaria* s.l. species into six sections according to the numbers of lobes at the apex of petal, styles, lobes at the apex of capsule and seeds per capsule, viz. sect. *Adenonema* (Bge.) Pax, sect. *Fimbripetalum* Turcz., sect. *Leucostemma* (Benth. ex G. Don f.) Pax, sect. *Oligosperma* Boiss., sect. *Schizotechium* Fenzl and sect. *Stellaria*. Recent molecular phylogenetic results changed the generic circumscription of the genus dramatically (Greenberg and Donoghue, 2011; Sharples and Tripp, 2019; Yao et al., 2021). Based on both molecular and morphological evidence as provided in Sharples and Tripp (2019), the traditionally circumscribed *Stellaria* s.l. in China should be at least divided into five genera: *Adenonema*, *Mesostemma*, *Nubelaria*, *Schizotechium* and *Stellaria* s.s.

Our results showed that the Chinese endemic species *Stellaria bistylata* (clade II-D; sampled with two accessions) from *S.* sect. *Oligosperma* is distantly related to *Stellaria* s.s. (clade I-C) (**Figure 4**). The species represents an independent lineage within Clade II and it is sister to a large group including the three subclades II-E (*Mesostemma*), II-F (comprising *Odontostemma*, *Pseudostellaria jamesiana*, and *Stellaria americana*) and II-G (*Pseudostellaria* except *P. jamesiana*, and *Shivparvatia*) with strong supports (BS = 94%, PP= 1.00; **Figure 4**). Morphologically, *S. bistylata* is mostly similar to *Mesostemma* in having shallowly 2-lobed petals (**Figure 3C**), 2 styles, 10 stamens, 4-valved capsules and 1–2 seeds per capsule (**Figure 3E**), but differs from the latter by having terminal dichotomous cymes (**Figures 3C, G**) (vs. paniculate inflorescence), five-merous flowers (**Figure 3C**) (vs. four-merous flowers), obovoid capsules (**Figure 3E**) (vs. ovoid capsules), and tuberculate surface of seeds (vs. corrugate surface of seeds). Additionally, the above-mentioned characters of petals, styles, capsules and seeds observed in *S. bistylata* can be also used to distinguish it easily from the currently circumscribed genus *Stellaria* s.s., which has deeply 2-lobed petals (**Figures 1H, I**), 3 (rarely 5) styles, 6 (rarely 10)-valved capsules and numerous seeds per capsule. Based on the consideration of morphological heterogeneity between *S. bistylata* and other genera of Alsineae (**Box 1**), and the currently revealed phylogenetic position of *S. bistylata*, we suggest that an independent status at the generic level for *S. bistylata* seems to be reasonable.

Additionally, the Chinese species *Stellaria delavayi* was placed in *S.* sect. *Schizotechium* based on morphological evidence (Wu and Ke, 1996), and in our molecular analyses it was nested within the genus *Schizotechium* with strong supports (BS = 100%, PP= 1.00; **Figure 4**). Therefore, both morphological and molecular evidence supported *Stellaria delavayi* should be transferred to *Schizotechium.*

### 4.4 Temporal diversification of Caryophyllaceae and the tribe Alsineae

Our study provides the first comprehensive tribe-level molecular dating analysis of Caryophyllaceae (**Figure 5**). The crown age (ca. 75.6 Ma; node 1 in **Figure 5**) of Caryophyllaceae estimated here is largely consistent with the timeframe of the earliest pollen fossil assigned to the family, that is *Periporopollenites polyoratus* (Couper) Stover & Partridge reported from the Late Campanian (Stover and Partridge, 1973; Jordan and Macphail, 2003). The split between the tribe Polycarpaeae and its sister clade (ca. 66.7 Ma; node 3) estimated here is largely consistent with that reported in Li et al. (2019; ca. 63.9 Ma), in which the two early diverged tribes (viz., Corrigioleae and Paronychieae) in Caryophyllaceae were not sampled. The stem age of the “higher Caryophyllaceae” (ca. 58.4 Ma; node 4) estimated here is also largely consistent with those reported in Li et al. (2019; ca. 53. 1 Ma) and Yao et al. (2019; 57.2 Ma).

Rapid radiation in the early diversification of the “higher Caryophyllaceae” may have occurred during the early Eocene (**Figure 5**), which is likely associated with the warm climate condition during the Early Eocene Climate Optimum (EECO) (Elson et al., 2022). The mean annual rainfall and temperature increased significantly during the early Eocene (**Figure 5B**), and these climate changes were suggested to have promoted a major increase in floral diversity (Wilf et al., 2003; Gayó et al., 2005; Zachos et al., 2008; Bondarenko and Utescher, 2023). Rapid diversifications occurred during the early Eocene were also reported in case studies of angiosperm families including Asteraceae (Huang et al., 2016), Cucurbitaceae (Guo et al., 2020) and Fagaceae (Zhou et al., 2022). Here, based on the dating analysis of Caryophyllaceae, we provided a new case study to understand not only the impact of the early Eocene climate change on the evolutionary history of angiosperms, but also the development history of the herbaceous flora in the northern temperate regions.

In Alsineae, most divergent events within the two major clades occurred since the late Oligocene (**Figure 5**), especially within clade II, the crown age of which was estimated here at ca. 28.1 Ma during the late Oligocene. Similar temporal evolutionary history was also reported in the early diversification of other angiosperm lineages, such as the phaseoloid legumes (Li et al., 2013), the Asian *Dendrobium* (Xiang et al., 2016), and the cacti family Cactaceae (Hernández-Hernández et al., 2014). Additionally, Gallaher et al. (2022) also suggested that the diversification of multiple extant lineages within the grass family Poaceae started during the similar time interval, such as Airinae (ca. 24.7 Ma), Arthropogoninae (ca. 28.1 Ma), Bambuseae (ca. 24.9 Ma), Ehrharteae (ca. 25.7 Ma), Tripogoninae (ca. 25.0 Ma) and Zizaniinae (ca. 25.0 Ma). The temporal concordance of these diversification events as revealed in different plant groups might be in response to the late Oligocene warming and aridity, which had been suggested for early diversification events of the phaseoloid legumes (Li et al., 2013). Thus we suggest that the special climate condition occurred during the late Oligocene may have triggered another episode for the global development of the herbaceous flora, as referred to the results from analysis of Alsineae (**Figure 5**) and also those from Gallaher et al. (2022).

Additionally, most inter-generic or infra-generic diversification events within Alsineae (such as in genera *Cerastium*, *Pseudostellaria*, *Odontostemma*, *Schivparvatia*, *Schizotechium* and *Stellaria*) occurred from middle to late Miocene (**Figure 5**). Similar temporal evolutionary history was also reported in genera belong to other tribes of Caryophyllaceae, including the genus *Acanthophyllum* C.A. Mey. in Caryophylleae (Mahmoudi-Shamsabad et al., 2021), *Gymnocarpos* Forssk. in Paronychieae (Jia et al., 2016), as well as *Heliosperma* (Rchb.) Rchb. (Frajman et al., 2009), *Lychnis* L. (Gizaw et al., 2016) and *Silene* L. (Petri et al., 2013) in Sileneae. This may be associated with the global climate change towards cooler and drier following the mid-Miocene Climate Optimum (MMCO; Holbourn et al., 2015). The globally cooler and drier climate following the mid-Miocene probably also promoted the extensive ecosystem expansion of C_4_ herbs (Sage et al., 2012) and the global development of herbaceous plants in Gnaphalieae (Nie et al., 2016), as well as the rapid radiation of some recent lineages within the grass family (Gallaher et al., 2022). Integrating the result obtained here from analysis of Alsineae, we suggest that the cooling and dry climatic condition since the mid-Miocene may also have a great impact on the evolution of the modern herbaceous flora. Nevertheless, an enlarged taxon sampling of Caryophyllaceae, especially an extensive sampling of the other 11 tribes, is still needed to better understand the evolutionary history of the large herbaceous family.

For some genera mainly distributed in Tibet-Himalaya-Hengduan region, i.e. *Odontostemma*, *Schivparvatia* and *Schizotechium*, speciation events within them mainly occurred since the middle to late Miocene (**Figure 5**), are probably also associated with the mountain uplifting events occurring in that period, which had been reported in multiple angiosperm lineages mainly distributed there, such as the alpine bamboos (Ye et al., 2018), *Cyananthus* Wall. ex Benth. (Zhou et al., 2013), *Isodon* (Schrad. ex Benth.) Spach (Yu et al., 2014), *Rheum* L. (Sun et al., 2012) and *Rhodiola* L. (Zhang et al., 2014). A well-resolved phylogenetic framework of Alsineae based on denser taxon sampling, especially an enlarged species-level sampling, is needed in further study in order to better understand the evolutionary history of the tribe.

## 5 Taxonomic treatments

### 5.1 Hesperostellaria

Gang Yao, B. Xue & Z.Q. Song, **gen. nov.** (**Figures 2B, E, F**)

**Type:** *Hesperostellaria americana* (Porter ex B.L. Rob.) Gang Yao, B. Xue & Z.Q. Song, **comb. nov.** ≡ *Stellaria dichotoma* var. *americana* Porter ex B.L. Rob., Proc. Amer. Acad. Arts 29: 289. 1894. ≡ *Alsine americana* (Porter ex B.L. Rob.) Rydb., Mem. New York Bot. Gard. 1: 144. 1900. ≡ *Stellaria americana* (Porter ex B.L. Rob.) Standl., Contr. U.S. Natl. Herb. 22(5): 336. 1921. ≡ *Arenaria stephaniana* var. *americana* (Porter ex B.L. Rob.) Shinners, Sida 1(1): 50. 1962. ≡ *Schizotechium americanum* (Porter ex B.L. Rob.) Arabi, Rabeler & Zarre, in Taxon 71(3): 624. 2022. Holotype: U.S.A., Montana, near Virginia City, 1871, *W.B. Platt* (*Hayden Survey*) *s.n.* [GH-00037985, photo!; isotypes: NY-00353059, PH-00027794 (**Figure 2B**), photos]!

**Diagnosis:** The new genus *Hesperostellaria* is most closely related to *Stellaria* in morphology, but it differs from the latter by having 5 stamens (vs. 10 stamens), 3-valved capsules (vs. 6- valved capsules, or rarely 5-valved capsules), and 3–6 seeds per capsule (vs. numerous seeds per capsule).

**Description:** Plants perennial, forming loose, prostrate mats, from rhizomatous rootstocks. Stems spreading, branched, very leafy, 4-angled. Leaves opposite, sessile; blade ovate to ovate-lanceolate, widest at or above middle, base round to cuneate, margins not scarious, apex usually obtuse, viscid. Inflorescences terminal, 1–5-flowered, very leafy cymes; bracts paired, foliaceous. Pedicels ca. 10 mm in flower, elongating, recurved and tortuous in fruit, pushing capsule into substrate. Flowers bisexual; perianth and androecium hypogynous; sepals 5, obscurely veined, ovate-obtuse, margins narrow, scarious; petals 5, slightly longer than sepals; stamens 5, filaments distinct; styles 3, ascending. Capsules broadly ovoid to globose, apex obtuse, tardily dehiscent with 3 valves; carpophore absent. Seeds 3–6, rusty brown, sub-ovate, finely tuberculate.

**Diversity:** Only one species, *Hesperostellaria americana* (Porter ex B.L. Rob.) Gang Yao, B. Xue & Z.Q. Song.

**Etymology:** This new genus is named after its western distribution and its morphological similarity to the genus *Stellaria*.

**Distribution:** The genus is endemic to western North America, and it grows usually on rocky slopes and talus, at the elevation of 1400–2800 m.

### 5.2 Reniostellaria

Gang Yao, B. Xue & Z.Q. Song, **gen. nov.** (**Figure 3**)

**Type:** *Reniostellaria bistylata* (W.Z. Di & Y. Ren) Gang Yao, B. Xue & Z.Q. Song, **comb. nov.** ≡ *Stellaria bistylata* W.Z. Di & Y. Ren, Acta Bot. Boreal.-Occid. Sin. 5(3): 231. 1985. Holotype: CHINA. Nei Meng-Gu (Inner Mongolia), the Helan Mountain, Halawugou, alt. 2390–2400 m, 27 July 1984, *EHNWU 6413* (WNU).

**Diagnosis:** The new genus *Reniostellaria* is most closely related to *Mesostemma* in morphology, but it differs from the latter by having terminal dichotomous cymes (vs. paniculate inflorescence), five-merous flowers (vs. four-merous flowers), obovoid capsules (vs. ovoid capsules), and tuberculate surface of seeds (vs. corrugate surface of seeds).

**Description:** Herbs, perennial. Stems densely tufted, diffuse, subterete, dichotomously branched, densely glandular hairy. Leaves succulent to some extent, oblong-lanceolate, base narrowed, apex acute. Flowers in terminal dichotomous cymes, bracts lanceolate, both surfaces glandular hairy. Sepals 5, oblong-lanceolate, outside shortly hairy or glabrous, margin broadly membranous, apex acute. Petals 5, obovate, apex shallowly 2-lobed. Stamens 10. Ovary globose, 1-loculed; ovules 4 or 5; Styles 2 (or rarely 3). Capsule obovoid, not exerted from the persistent sepals, apex 4(or rarely 6)-valved. Seeds 1 (or 2), black-brown, ovoid to obovoid, tuberculate.

**Diversity:** Only one species, *Reniostellaria bistylata* (W.Z. Di & Y. Ren) Gang Yao, B. Xue & Z.Q. Song.

**Etymology:** This new genus is named in honor of the Chinese botanist Yi Ren (1959–2019), who firstly described the species *Stellaria bistylata* (= *Reniostellaria bistylata*).

**Distribution:** The genus is narrowly restricted in valleys of the Helan Mountain at the border of Ningxia and Inner Mongolia, China. It grows usually in dry gullies, at the elevation of (1400) 2000–2800 m.

**Taxonomic note:** The two names *Stellaria bistylata* W.Z. Di & Y. Ren and *S. bistyla* Y.Z. Zhao were published in the same year, 1985, with the latter name being clearly recorded to be published in October (Zhao, 1985). The former name *S. bistylata* was published in *Acta Botanica Boreali-Occidentalia Sinica* 5(3) (Ren and Di, 1985), in which the date of publication was not clearly indicated. But there was a statement in the last issue of 1984, which indicated each of the third issue after 1984 would be published on 1 September. It was further confirmed by the fact that the third issue of the sixth volume of the journal bears the date September 1986. Thus, September 1985 should be the publication date for *S. bistylata*, which has priority over *S. bistyla* according to Article 11.4 of *ICN* (Turland et al., 2018), although *S. bistyla* was accepted by some authors (Wu, 1991; Wu and Ke, 1996; Lu et al., 2001). In the present study, although the holotype (*EHNWU 6413*, WNU) of *S. bistylata* was not available to us, we examined multiple of its paratypes deposited in the herbarium WUK, one of which (*Y.Q. He 7699*, WUK-0156150, photo)! was collected in the same locality as the holotype with linear drawing and morphological description of flowers. Our examination of specimens and original descriptions of both *S. bistylata* and *S. bistyla* also confirmed that the two names represent the same species.

### 5.3 Torreyostellaria

Gang Yao, B. Xue & Z.Q. Song, **gen. nov.** (**Figures 2A, C, D**)

**Type**: *Torreyostellaria jamesiana* (Torr.) Gang Yao, B. Xue & Z.Q. Song, **comb. nov.** ≡ *Stellaria jamesiana* Torr., Ann. Lyceum Nat. Hist. New York 2(6): 169. 1827. ≡ *Arenaria jamesiana* (Torr.) Shinners, Sida 1(1): 50. 1962. ≡ *Pseudostellaria jamesiana* (Torr.) W.A. Weber & R.L. Hartm., Phytologia 44(4): 314. 1979. ≡ *Schizotechium jamesianum* (Torr.) Arabi, Rabeler & Zarre, in Taxon 71(3): 625. 2022. Holotype: U.S.A., within the Rocky Mountains, *E. James s.n.* (NY-00353063, photo!; **Figure 2A**).

**Diagnosis:** The new genus *Torreyostellaria* is closely related to *Pseudostellaria* and *Stellaria* in morphology. It is distinct from *Pseudostellaria* in having only chasmogamic flowers (vs. chasmogamic and cleistogamous flowers), 3 styles (vs. 2 styles), 6-valved capsules (vs. 3-valved or rarely 2- or 4-valved capsules), and large chromosome number (2n = 96 vs. 2n = 32). *Torreyostellaria* is morphologically distinguished from *Stellaria* by its tuberous root (vs. non-tuberous), shallowly 2-lobed petals (vs. deeply lobed petals) and 3–6 seeds per capsule (vs. numerous seeds per capsule).

**Description:** Herbs, perennial; taproots absent, rhizomes with spherical or elongate tuberous thickenings. Stems 4-angled, glabrous or stipitate-glandular throughout or at least in inflorescence, often densely so. Leaves opposite, sessile; blade 1-veined, linear to linear-lanceolate or broadly lanceolate, margins flat to briefly revolute, apex acute. Inflorescences open cymes, flowers often proliferating with age; bracts paired. Pedicels recurved to reflexed from base in fruit. Flowers: perianth and androecium hypogynous; sepals 5, distinct, lanceolate to narrowly ovate; petals evidently longer than sepals, apex lobed, lobes broadly rounded; anthers 10, arising from base of ovary; styles 3; stigmas terminal. Capsules ovoid, 6-lobed at apex. Seeds 1–3, red-brown or brown, circular to oblong or elliptic, plump or laterally compressed, tuberculate, marginal wing absent, appendage absent. 2n = 96.

**Diversity:** Only one species, *Torreyostellaria jamesiana* (Torr.) Gang Yao, B. Xue & Z.Q. Song.

**Etymology:** This new genus is named in honor of American botanist John Torrey (1796–1873), who firstly described the species *Stellaria jamesiana* (*= Torreyostellaria jamesiana*).

**Distribution:** The new genus is endemic to Western United States, and it grows usually in meadows, sagebrush-grasslands, dry understory of aspen and coniferous forests, at the elevation of 600–3400 m.

### 5.4 Schizotechium delavayi

(Franch.) Gang Yao, B. Xue & Z.Q. Song, **comb. nov. ≡** *Stellaria delavayi* Franch., Pl. Delavay. 97. 1889. Lectotype (designated here): CHINA. Yunnan Province, Coteaux de Huang li pin sur Ta-pin-tze, 22 September 1887, *J.M. Delavay 3131* (P-01902907; isolectotypes: K-000723437, K-000723438, P-01902906, P-01902908, P-01902909, P-01902910).

## Data availability statement

The datasets presented in this study can be found in online repositories. The names of the repository/repositories and accession number(s) can be found in the article/**Supplementary Material**.

## Author contributions

BX and GY participated in the conception and design of the research. BX, GY and JC did fieldwork. BX, ZM, JH, YL and GY are responsible for analyzing and processing data. BX, ZS and GY wrote the manuscript. The paper was revised by JC, ZM, JH and YL. All authors contributed to the article and approved the submitted version.

## Supplementary material

Supplementary Figure 1 Bayesian consensus tree obtained from analysis of the Caryophyllaceae-wide nrITS dataset. Posterior probability (PP) in Bayesian inference (BI) and bootstrap (BS) value in Maximum likelihood (ML) analysis are indicated above and below the stem branch of each phylogenetic node, respectively. NP indicates the topology was not present in BI analysis. The crown nodes of Alsineae and Caryophyllaceae are shown by the arrowheads.

Supplementary Figure 2 Maximum likelihood (ML) tree of Caryophyllaceae obtained from analysis of the Caryophyllaceae-wide cpDNA dataset (including *matK*, *rbcL*, *rps16* intron and *trnL-F* intergenic region). Posterior probability (PP) in Bayesian inference (BI) and bootstrap (BS) value in ML analysis are indicated above and below the stem branch of each phylogenetic node, respectively. NP indicates the topology was not present in BI analysis. The crown nodes of Alsineae and Caryophyllaceae are shown by the arrowheads.

Supplementary Figure 3 Maximum likelihood (ML) tree of Caryophyllaceae obtained from analysis of the Caryophyllaceae-wide cpDNA-nrITS dataset (including nrITS, *matK*, *rbcL*, *rps16* intron and *trnL-F* intergenic region). Posterior probability (PP) in Bayesian inference (BI) and bootstrap (BS) value in ML analysis are indicated above and below the stem branch of each phylogenetic node, respectively. NP indicates the topology was not present in BI analysis. The crown nodes of Alsineae and Caryophyllaceae are shown by the arrowheads.

Supplementary Figure 4 Maximum likelihood (ML) tree of Alsineae inferred from the reduced Alsineae-wide dataset (including nrITS, *matK*, *rbcL*, *rps16* intron and *trnL-F* intergenic region). Posterior probability (PP) in Bayesian inference (BI) and bootstrap (BS) value in ML analysis are indicated above and below the stem branch of each phylogenetic node, respectively. NP indicates the topology was not present in BI analysis. The crown node of Alsineae is shown by the arrowhead.
