# Supplementary material for: Phylogenetic analysis and temporal diversification of the tribe Alsineae (Caryophyllaceae) with the description of three new genera, Hesperostellaria, Reniostellaria and Torreyostellaria
Source: Front Plant Sci. 2023 Jun 21;14:1127443. doi: 10.3389/fpls.2023.1127443 (PMC10321415; doi:10.3389/fpls.2023.1127443)
Supplement: Supplementary Figure 1 — Bayesian consensus tree obtained from analysis of the Caryophyllaceae-wide nrITS dataset. Posterior probability (PP) in Bayesian inference (BI) and bootstrap (BS) value in Maximum likelihood (ML) analysis are indicated above and below the stem branch of each phylogenetic node, respectively. NP indicates the topology was not present in BI analysis. The crown nodes of Alsineae and Caryophyllaceae are shown by the arrowheads. [file DataSheet_1.zip › Supplementary Material Presentation/Supplementary Table S1.docx]

**Supplementary Material-Table S1** Sequences information for all samples used in the present study. Sequences newly generated in this study are marked in bold. Some taxa have two accession numbers under the maker ‘*trnL-F*’, indicating that the entire ‘*trnL-F*’ was disintegrated as two smaller sections in sequencing process in previous studies that producing these sequences, and then the two sections were combined in phylogenetic analysis.

| Taxa | GenBank accession numbers | | | | |
| --- | --- | --- | --- | --- | --- |
|  | *matK* | *rbcL* | *rps16* intron | *trnL-F* | nrITS |
| *Adenonema cherleriae* (Fisch. ex Ser.) M.T.Sharples & E.Tripp | **OP617346** | **OP617351** | **OP617356** | **OP617361** | **OP626174** |
| *Brachystemma calycinum* D.Don | **OP617347** | **OP617355** | **OP617358** | **OP617365** | **OP626178** |
| *Cerastium arvense* L. | AY936295 | JX848446 | MH243535 | FJ404976 | MH219805 |
| *Cerastium beeringianum* Cham. & Schltdl. | KC474448 | KC482422 | — | AY521318, AY521365 | MG236459 |
| *Cerastium davuricum* Fisch. ex Spreng. | KX158358 | KX158395 | KX158432 | — | KX158321 |
| *Cerastium dichotomum* L. subsp. *inflatum* (Link) Cullen | KX158359 | KX158396 | KX158433 | — | KX158322 |
| *Cerastium dinaricum* Beck & Szyszył. | — | — | — | KJ716526 | KJ716515 |
| *Cerastium falcatum* (Ser.) Bunge | — | — | MH243537 | AY521334, AY521382 | MH219807 |
| *Cerastium fontanum* Baumg. | KX821263 | KF602216 | FJ404899 | FJ404977 | GU444015 |
| *Cerastium furcatum* Cham. & Schltdl. | MH116578 | MH116103 | — | — | MH117479 |
| *Cerastium glomeratum* Thuill. | JN895359 | HM849882 | — | KY697436 | AY857977 |
| *Cerastium jiuhuashanense* Gang Yao et. J.W. Zhai | MT507771 | MT507771 | MT507771 | MT507771 | MT791125 |
| *Cerastium latifolium* L. | — | KF602212 | — | AY521301, AY521348 | — |
| *Cerastium nutans* Raf. | MK519892 | MK525532 | MT624650 | AY521339, AY521387 | MT624569 |
| *Cerastium nigrescens* (H.C.Watson) Edmondston ex H.C.Watson | — | KF997275 | — | AY521315, AY521362 | KX165939 |
| *Cerastium pusillum* Ser. | JN589226 | — | — | JN589683 | JN589112 |
| *Cerastium regelii* Ostenf. | KC474450 | KC482424 | — | AY521317, AY521364 | MG236500 |
| *Cerastium subtriflorum* Dalla Torre & Sarnth. | — | — | — | KJ716527 | MH537035 |
| *Cerastium szechuense* F.N. Williams | — | — | — | JN589674 | JN589116 |
| *Cerastium tomentosum* L. | JN589244 | KF997321 | MH243538 | AY521310, AY521357 | JN589031 |
| *Dichodon cerastoides* (L.) Rchb. | — | MG249356 | MH243542 | AY521340, AY521388 | MH219812 |
| *Dichodon dubium* (Bastard) Ikonn. | — | — | MH243544 | AY521341, AY521389 | MH219815 |
| *Hartmaniella oxyphylla* (B.L. Rob.) M.L. Zhang | KX158348 | KX158385 | KX158422 | — | KX158311 |
| *Hartmaniella sierra* (Rabeler & R.L. Hartm.) M.L. Zhang | KX158351 | KX158388 | KX158425 | — | KX158314 |
| *Holosteum marginatum* C.A. Mey. | JN589261 | — | — | JN589732 | JN589093 |
| *Holosteum umbellatum* L. | MK520188 | MK525977 | FJ404909 | JN589655 | JN589051 |
| *Lepyrodiclis holosteoides* (C. A. Mey.) Fisch. et Mey. | FJ404840 | JQ933385 | KP149043 | FJ404989 | MH808295 |
| *Mesostemma platyphyllum* (Rech.f.) Rech.f. | — | — | MT624666 | — | MT624584 |
| *Mesostemma perfoliatum* (Rech.f.) Rech.f. | — | — | MT624665 | — | MT624583 |
| *Mesostemma kotschyanum* (Fenzl ex Boiss.) Vved. | — | — | MT624664 | — | MT624582 |
| *Moenchia erecta* (L.) G. Gaertn., B. Mey. & Scherb. | JN895271 | JN892479 | FJ404926 | FJ405002 | JN589103 |
| *Nubelaria arisanensis* (Hayata) M.T.Sharples & E.A.Tripp | JN589258 | MT384967 | — | — | JN589096 |
| *Nubelaria diversiflora* (Maxim.) M.T.Sharples & E.A.Tripp | JN589235 | — | MT624669 | LC568827 | JN589119 |
| *Odontostemma barbatum* (Franch.) Sadeghian & Zarre | — | — | — | — | KP148852 |
| *Odontostemma fridericae* (Hand.-Mazz.) Sadeghian & Zarre | — | — | — | — | AY936332 |
| *Odontostemma leucasterium* (Mattf.) Rabeler & W.L. Wagner | MK341306 | — | MK341256 | MK341198 | MK341342 |
| *Odontostemma melanandrum* (Maxim.) Rabeler & W.L. Wagner | MK341378 | — | MK341255 | MK341197 | MK341343 |
| *Odontostemma roseiflorum* (Sprague) Sadeghian & Zarre | FJ404825 | — | FJ404895 | FJ404971 | AY936244 |
| *Odontostemma saginoides* (Maxim.) Rabeler & W. L. Wagner | — | — | — | — | MT791129 |
| *Odontostemma spathulifolium* (C. Y. Wu ex L. H. Zhou) Rabeler & W. L. Wagner | — | — | — | — | MT791131 |
| *Odontostemma trichophorum* (Franch.) Sadeghian & Zarre | — | — | — | — | AY936243 |
| *Pseudostellaria jamesiana* (Torr.) W.A. Weber & R.L. Hartm._ZXQ_8 | KX158343 | KX158380 | KX158417 | — | KX158306 |
| *Pseudostellaria jamesiana* (Torr.) W.A. Weber & R.L. Hartm. | FJ404861 | MF963180 | FJ404933 | FJ405010 | JN589048 |
| *Pseudostellaria japonica* (Korsh.) Pax | KX158344 | KX158381 | KX158418 | KY063323 | KX158307 |
| *Pseudostellaria maximowicziana* (Franch. & Sav.) Pax | KX158346 | KX158383 | KX158420 | — | KX158309 |
| *Pseudostellaria tianmushanensis* G.H. Xia & G.Y. Li | KX158355 | KX158392 | KX158429 | — | KX158318 |
| *Pseudostellaria tibetica* Ohwi *_ZXQ_21* | KX158347 | KX158384 | KX158421 | — | KX158310 |
| *Pseudostellaria tibetica* Ohwi *_ZXQ_22* | KX158354 | KX158391 | KX158428 | — | KX158317 |
| *Pseudostellaria davidii* (Franch.) Pax *_ZXQ_1* | KX158338 | KX158375 | KX158412 | KY063300 | KX158301 |
| *Pseudostellaria ebracteata* (Kom.) N.S.Pavlova*_ZXQ_3* | KX158340 | KX158377 | KX158414 | — | KX158303 |
| *Pseudostellaria europaea* Schaeftl.*_ZXQ_4* | KX158356 | KX158393 | KX158430 | — | KX158319 |
| *Pseudostellaria_heterantha* (Maxim.) Pax*_ZXQ_5* | KX158341 | KX158378 | KX158415 | — | KX158304 |
| *Pseudostellaria_rigida* (Komarov) Pax*_ZXQ_15* | KX158349 | KX158386 | KX158423 | — | KX158312 |
| *Pseudostellaria rupestris* (Turcz.) Pax*_ZXQ_16* | KX158350 | KX158387 | KX158424 | — | KX158313 |
| *Pseudostellaria sylvatica* (Maxim.) Pax*_ZXQ_18* | KX158352 | KX158389 | KX158426 | KY063325 | KX158315 |
| *Pseudostellaria longipedicellata* S.Lee, K.I.Heo & S.C.Kim | NC_039454 | NC_039454 | NC_039454 | NC_039454 | KY063140 |
| *Pseudostellaria okamotoi* Ohwi | NC_039974 | NC_039974 | NC_039974 | NC_039974 | KY063147 |
| *Pseudostellaria setulosa* Ohwi | NC_041462 | NC_041462 | NC_041462 | NC_041462 | KY063182 |
| *Pseudostellaria palibiniana* (Takeda) Ohwi | NC_041166 | NC_041166 | NC_041166 | NC_041166 | KY078450 |
| *Pseudostellaria heterophylla* (Miq.) Pax | NC_044183 | NC_044183 | NC_044183 | NC_044183 | MH808298 |
| *Rabelera holostea* (L.) M.T.Sharples & E.Tripp | KX183916 | FJ395575 | MH243549 | JN589664 | KX183997 |
| *Schizotechium paniculatum* (Edgew.) Pusalkar & S.K.Srivast. | **OP617350** | **OP617353** | **OP617360** | **OP617362** | **OP626176** |
| *Schizotechium monospermum* (Buch.-Ham. ex D.Don) Pusalkar & S.K. Srivast. | JN589243 | — | — | — | JN589153 |
| *Shivparvatia ciliolata* (Edgew.) Pusalkar & D. K. Singh | — | — | — | — | KP148859 |
| *Shivparvatia forrestii* (Diels) Rabeler | MK341374 | — | MK341251 | MK341193 | MK341340 |
| *Shivparvatia glanduligera* (Edgew.) Pusalkar & D.K.Singh | MK341375 | — | MK341252 | MK341194 | MK341341 |
| *Shivparvatia melandryoides* (Edgew.) Satish Chandra and D. S. Rawat | — | — | — | — | MT791126 |
| *Shivparvatia stracheyi* (Edgew.) Pusalkar & D. K. Singh | — | — | — | — | KP148898 |
| *Stellaria alsine* Grimm | HM850778 | HM850385 | — | EU785987 | AY438312 |
| *Stellaria aquatic* (L.) Scop. | JN894058 | KM360890 | MH243547 | FJ405004 | AY594303 |
| *Stellaria americana* (Porter ex B.L. Rob.) Standl._ ZXQ-34 | KX158372 | KX158409 | KX158446 | — | KX158335 |
| *Stellaria americana* (Porter ex B.L. Rob.) Standl. | JN589202 | — | — | JN589675 | JN589090 |
| *Stellaria bistylata* W. Zh. Di et Y. Ren | MK534858 | MK534811 | — | — | MK555247 |
| *Stellaria bistylata* W. Zh. Di et Y. Ren_YG208 | **OP617348** | **OP617354** | **OP617357** | **OP617364** | **OP626175** |
| *Stellaria borealis* Bigelow | JN589285 | MG247728 | — | JN589713 | JN589064 |
| *Stellaria chinensis* Regel | JN589241 | — | — | EU785990 | JN589133 |
| *Stellaria corei* Shinners | JN589300 | — | — | JN589715 | JN589046 |
| *Stellaria crassifolia* Ehrh. | KC475924 | KC484145 | — | JN589701 | JN589071 |
| *Stellaria cuspidata* Willd. ex D.F.K. Schltdl. | JN589268 | — | FJ404952 | JN589641 | JN589099 |
| *Stellaria delavayi* Franch | **OP617349** | **OP617352** | **OP617359** | **OP617363** | **OP626177** |
| *Stellaria graminea* L. | MK520714 | KM360998 | MH243548 | JN589687 | AY594304 |
| *Stellaria longifolia* Muhl. ex Willd. | MK520715 | JX848448 | — | GQ245567 | JN589146 |
| *Stellaria longipes* Goldie | KC475949 | JX848449 | — | JN589672 | JN589086 |
| *Stellaria media* (L.) Vill. | HM850779 | AF206823 | Z83152 | EU785989 | MK044722 |
| *Stellaria nemorum* L. | AY936298 | JN893484 | — | HM590349 | AY936246 |
| *Stellaria palustris* Ehrh. ex Retz. | MK520716 | KX158401 | KX158438 | — | JN589080 |
| *Stellaria pentastyla* W.Qiao Wang, H.F. Xu & Z.H. Ma_SC0054 | MN892512 | — | — | MN892507 | MN879262 |
| *Stellaria pentastyla* W.Qiao Wang, H.F. Xu & Z.H. Ma_YN0014 | MN892511 | — | — | MN892506 | MN879261 |
| *Stellaria pubera* Michx. | FJ404878 | KP643834 | — | FJ405027 | JN589127 |
| *Stellaria soongorica* Roshev. | MF158660 | KX158402 | KX158439 | — | KX158328 |
| *Stellaria umbellata* Turcz. | JN589254 | MG246195 | — | JN589737 | JN589109 |
| *Stellaria vestita* Kurz | MH116882 | MH116433 | — | EU785988 | MH117776 |
| **Outgroups** |  |  |  |  |  |
| *Arenaria serpyllifolia* L. | KX158357 | KX158394 | KX158431 | FJ404972 | KX158320 |
| *Arenaria lanuginose* (Michx.) Rohrb. | FJ404821 | MH028838 | FJ404891 | FJ404968 | KP148878 |
| *Corrigiola litoralis* L. | FN825767 | FN868311 | FJ404902 | FJ404979 | HE602452 |
| *Telephium imperati* L. | FN825768 | FN868312 | FJ404955 | JN589742 | JN589092 |
| *Herniaria glabra* L. | MK926048 | AF132091 | KY513562 | JN589730 | AJ310965 |
| *Paronychia kapela* A.Kern. | AY936284 | — | KY513568 | KY616130 | AJ310967 |
| *Gymnocarpos decandrus* Forssk. | FJ404837 | MT645209 | KX012950 | KY616122 | KX012968 |
| *Drymaria* | MH551956 | KJ773466 | FJ404905 | FJ404983 | FJ980408 |
| *Pycnophyllum* | FJ460219 | DQ267194 | FJ404934 | FJ405011 | JN589108 |
| *Loeflingia* | AY936288 | MF963375 | AM501419 | JN589659 | MF964124 |
| *Spergularia marina* | KY952512 | HM850381 | KY513599 | KY616160 | MH808307 |
| *Spergula arvensis* L. | JN589292 | KM360994 | KY513576 | KY616142 | JX274532 |
| *Eremogone baxoiensis* (L.H.Zhou) Dillenb. & Kadereit | MK341380 | — | MK341258 | MK341199 | MK341313 |
| *Eremogone capillaris* (Poir.) Fenzl | KC474711 | MG249014 | KP149011 | GQ244597 | KP148909 |
| *Eremogone acicularis* (F.N.Williams ex Keissl.) Ikonn. | MK341301 | — | MK341286 | MK341227 | MK341309 |
| *Arenaria densissima* Wall. | MK341362 | — | MK341232 | MK341174 | MK341345 |
| *Arenaria oreophila* Hook.f. | MK341298 | — | MK341245 | MK341187 | MK341356 |
| *Thylacospermum caespitosum* (Cambess.) Schischk. | MK341373 | — | MK341250 | MK341192 | MK341359 |
| *Agrostemma githago* L. | FJ589503 | KM360618 | Z83154 | JN589684 | JN589107 |
| *Heliosperma* | FJ589506 | — | LC423845 | JN589653 | LC424045 |
| *Silene aprica* Turcz. | MH658952 | KX158399 | LC423907 | FN821322 | MK555254 |
| *Atocion* | FJ589504 | HG417049 | FJ376820 | KY697498 | FJ384033 |
| *Schiedea membranacea* H.St.John | DQ907816 | DQ907762 | GQ224312 | FJ405015 | AY517662 |
| *Mononeuria glabra* (Michx.) Dillenb. & Kadereit | HQ235325 | HQ235608 | AY727451 | AY727206, AY727239 | MK355977 |
| *Scleranthus annuus* L. | FJ404869 | AY270145 | FJ404943 | JN589638 | JX274538 |
| *Sagina* | KF737569 | KF997390 | FJ404935 | JN589717 | MH808303 |
| *Colobanthus* | FJ404830 | JQ933274 | FJ404901 | JN589705 | MH781182 |
| *Bufonia* | FJ404827 | KX709610 | FJ404897 | FJ404974 | JN589044 |
| *Moehringia* | FJ404851 | KM360884 | FJ404924 | FJ405000 | JX274536 |
| *Psammosilene tunicoides* W.C.Wu & C.Y.Wu | NC_045947 | NC_045947 | NC_045947 | NC_045947 | JN589122 |
| *Dianthus caryophyllus* L. | NC_039650 | NC_039650 | NC_039650 | NC_039650 | KU722881 |
| *Acanthophyllum* | JN589209 | — | MN310752 | JN589663 | JN589016 |
| *Gypsophila vaccaria* (L.) Sm. | NC_040936 | NC_040936 | NC_040936 | NC_040936 | MH711400 |
| *Chenopodium album* Bosc. ex Moq. | MW417304 | MW417304 | MW417304 | MW417304 | MN396437 |
| *Amaranthus hybridus* L. | NC_053787 | NC_053787 | NC_053787 | NC_053787 | MT811921 |
| *Salicornia brachiate* Miq. | NC_027224 | NC_027224 | NC_027224 | NC_027224 | KF848296 |
| *Bienertia sinuspersici* Akhani | KU726550 | KU726550 | KU726550 | KU726550 | DQ499349 |
| *Achatocarpus nigricans* Triana | MK397908 | MK397908 | MK397908 | MK397908 | — |
| *Phaulothamnus spinescens* A.Gray | MH286322 | MH286322 | MH286322 | MH286322 | — |
| *Macarthuria keigheryi* Lepschi | MK397926 | MK397926 | MK397926 | MK397926 | — |
