# Supplementary figures and images for: Phylogenetic analysis and temporal diversification of the tribe Alsineae (Caryophyllaceae) with the description of three new genera, Hesperostellaria, Reniostellaria and Torreyostellaria
Source: Front Plant Sci. 2023 Jun 21;14:1127443. doi: 10.3389/fpls.2023.1127443 (PMC10321415; doi:10.3389/fpls.2023.1127443)

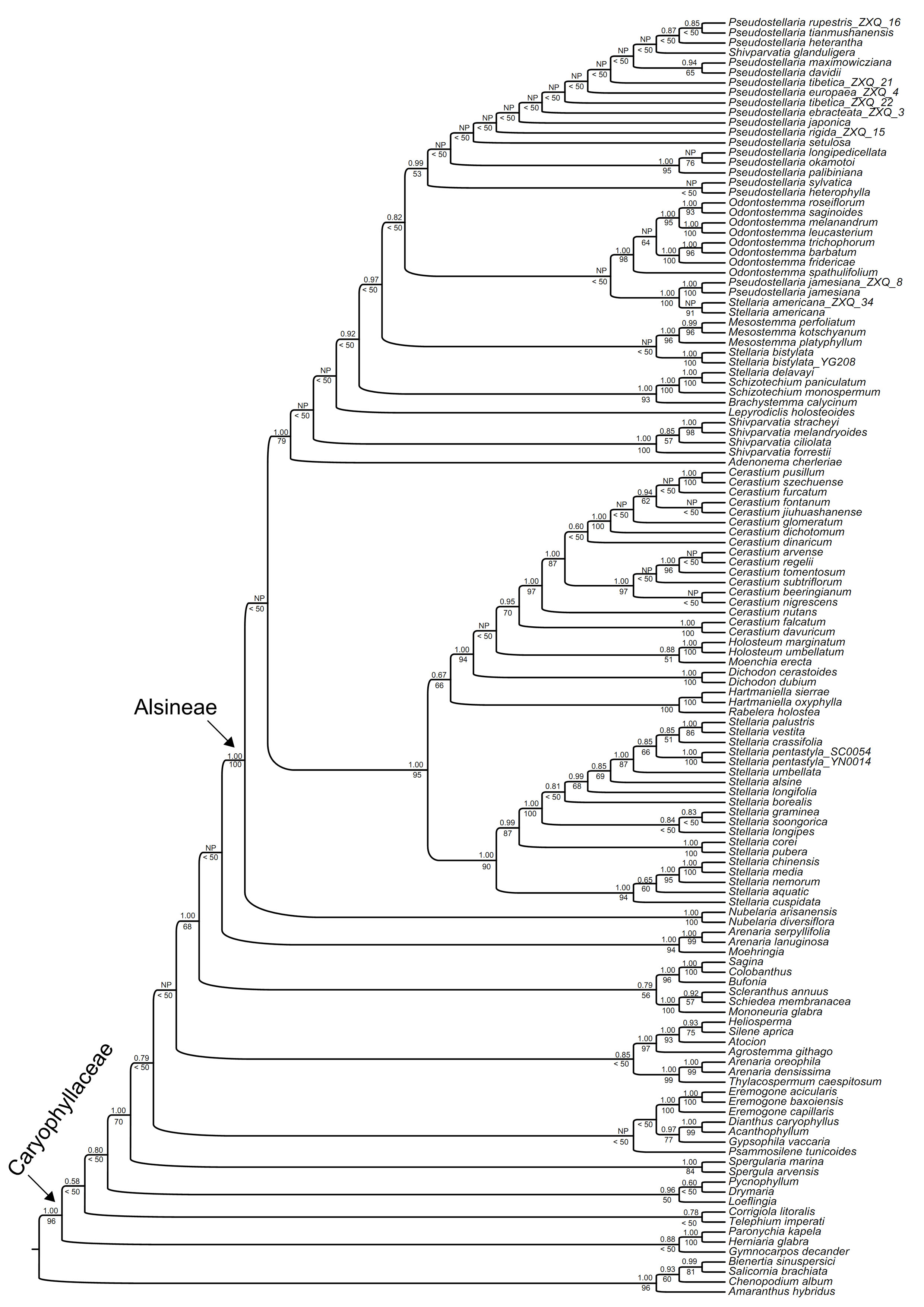

Supplement: Supplementary Figure 1 — Bayesian consensus tree obtained from analysis of the Caryophyllaceae-wide nrITS dataset. Posterior probability (PP) in Bayesian inference (BI) and bootstrap (BS) value in Maximum likelihood (ML) analysis are indicated above and below the stem branch of each phylogenetic node, respectively. NP indicates the topology was not present in BI analysis. The crown nodes of Alsineae and Caryophyllaceae are shown by the arrowheads. [file DataSheet_1.zip › Supplementary Material Presentation/Supplementary Figure S1.jpg]

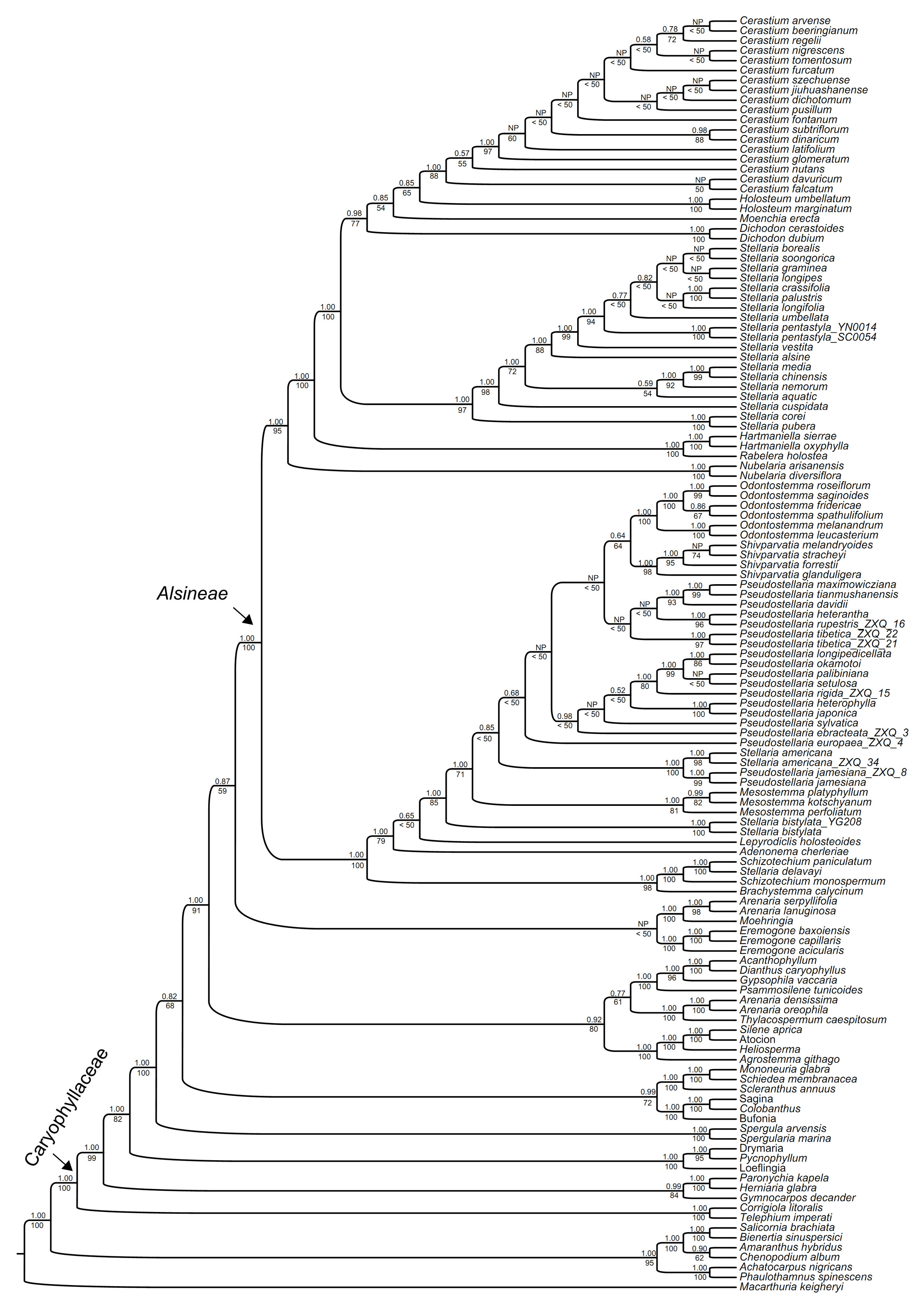

Supplement: Supplementary Figure 1 — Bayesian consensus tree obtained from analysis of the Caryophyllaceae-wide nrITS dataset. Posterior probability (PP) in Bayesian inference (BI) and bootstrap (BS) value in Maximum likelihood (ML) analysis are indicated above and below the stem branch of each phylogenetic node, respectively. NP indicates the topology was not present in BI analysis. The crown nodes of Alsineae and Caryophyllaceae are shown by the arrowheads. [file DataSheet_1.zip › Supplementary Material Presentation/Supplementary Figure S2.jpg]

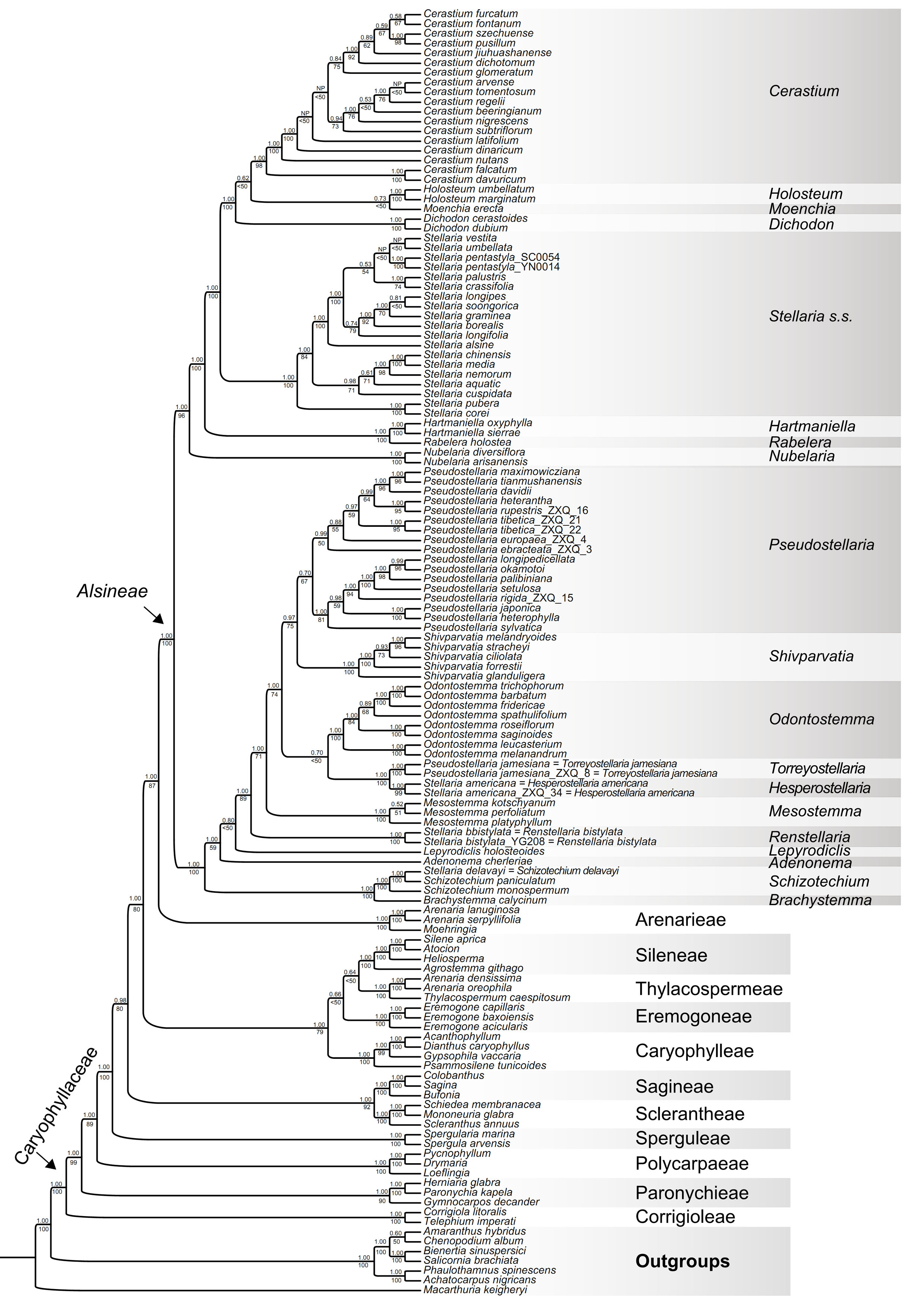

Supplement: Supplementary Figure 1 — Bayesian consensus tree obtained from analysis of the Caryophyllaceae-wide nrITS dataset. Posterior probability (PP) in Bayesian inference (BI) and bootstrap (BS) value in Maximum likelihood (ML) analysis are indicated above and below the stem branch of each phylogenetic node, respectively. NP indicates the topology was not present in BI analysis. The crown nodes of Alsineae and Caryophyllaceae are shown by the arrowheads. [file DataSheet_1.zip › Supplementary Material Presentation/Supplementary Figure S3.jpg]

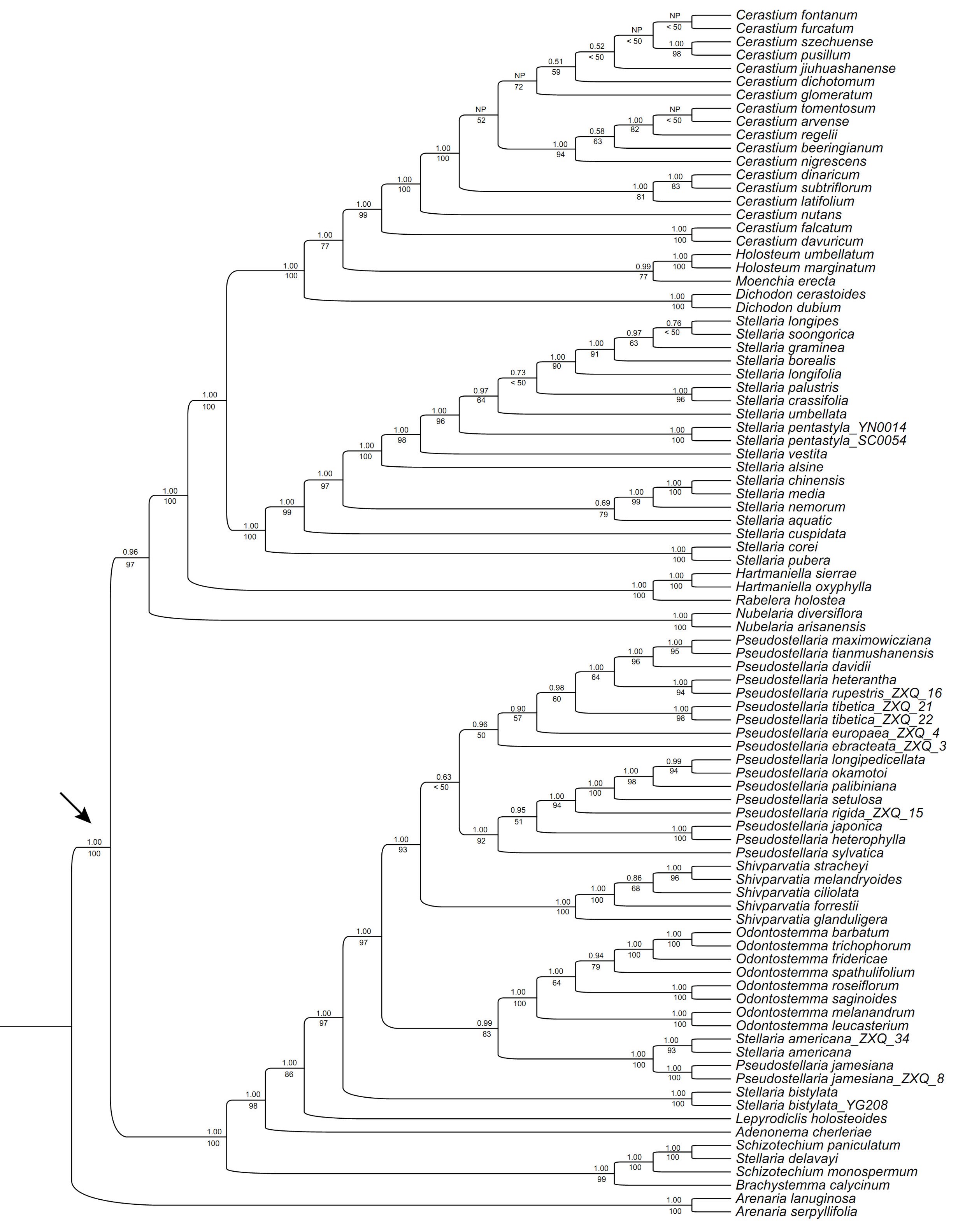

Supplement: Supplementary Figure 1 — Bayesian consensus tree obtained from analysis of the Caryophyllaceae-wide nrITS dataset. Posterior probability (PP) in Bayesian inference (BI) and bootstrap (BS) value in Maximum likelihood (ML) analysis are indicated above and below the stem branch of each phylogenetic node, respectively. NP indicates the topology was not present in BI analysis. The crown nodes of Alsineae and Caryophyllaceae are shown by the arrowheads. [file DataSheet_1.zip › Supplementary Material Presentation/Supplementary Figure S4.jpg]
